# Metabolomic profiling of renal cell carcinoma to identify prognostic markers of metastasis

**DOI:** 10.1371/journal.pone.0361404

**Published:** 2026-11-11

**Authors:** Michael Williams, Aditya Chakraborty, Brian Main, Liang Li, Ya-Chun Chan, Xiaohang Wang, Nico Verbeeck, Liam Spiers, Olivia Routten, Theodore Chin, Kaitlyn Bejar, Dean Troyer

**Affiliations:** 1 Urology of Virginia, Virginia Beach, and Department of Urology, Virginia Health Sciences at Old Dominion University, Norfolk, Virginia, United States of America; 2 Joint School of Public Health, Old Dominion University and Norfolk State University, Norfolk, Virginia, United States of America; 3 Leroy T. Canoles Jr. Cancer Research Center and Office of Research, Norfolk, Virginia, United States of America; 4 Department of Chemistry, University of Alberta, Edmonton, Alberta, Canada; 5 Aspect Analytics NV, Genk, Belgium; 6 Virginia Health Sciences at Old Dominion University, Norfolk, Virginia, United States of America; 7 Office of Research, Virginia Health Sciences at Old Dominion University, Norfolk, Virginia, United States of America; 8 Cell Systems and Anatomy, UT Health San Antonio, Texas, United States of America; 9 Leroy T. Canoles Jr. Cancer Research Center and Dept of Biological and Translational Sciences, Virginia Health Sciences at Old Dominion University, Norfolk, Virginia, United States of America; 10 Adjunct Professor, Dept of Pathology, UT Health San Antonio, San Antonio, Texas, United States of America; University of Westminster - Regent Street Campus: University of Westminster, UNITED KINGDOM OF GREAT BRITAIN AND NORTHERN IRELAND

## Abstract

**Background:**

Patients with clear cell renal cell carcinoma (ccRCC) undergo surveillance for recurrent or metastatic disease following nephrectomy. The primary aim of this study was identification of prognostic biomarkers for metastatic disease.

**Methods:**

We conducted a retrospective case control study of ccRCC patients treated with surgical resection. At 3 years follow up tumors were classiﬁed as cases or non-metastatic tumors based on presence or absence of metastases. Fresh frozen ccRCC tumors and adjacent normal tissues were analyzed by combining a chemical labeling method with liquid chromatography-mass spectrometry (LC-MS). Three different chemical groups were analyzed: Carboxyl, Amine-Phenol, and Hydroxyl. Metabolites were identiﬁed using a chemical library for cross-reference. LC-MS results were analyzed with machine learning approaches and statistical models. Unsupervised machine learning (UMAP, Non-negative Matrix Factorization) was used for exploratory analysis of the data. Supervised machine learning, using both linear and nonlinear Support Vector Machines (SVM), was used to construct classiﬁcation models. Repeated cross-validation was used to avoid overﬁtting and gain representative classiﬁcation performance of the model.

**Results:**

Machine learning and SVM classification models showed good classiﬁcation performance with an area under the Precision-Recall curve (AUPRC) of 0.92 for the best-performing model. Metabolites showing statistically significant changes associated with metastasis included the following: 4-Hydroxybutanoic acid, Glutamyl-Glutamic acid, 3-Aminosalicylic acid, Indoxyl sulfate, 3,4-Dihydroxyphenylpropanoic acid, and N-ethylglycine.

**Conclusions:**

Metabolites which are prognostic for ccRCC metastasis were identified. Impact: Prognostic biomarkers could inform the frequency and intensity of post-operative surveillance of ccRCC. Identiﬁcation of such biomarkers could aid clinicians in the management of patients with ccRCC.

## Introduction

Cancer and metabolic alterations are closely linked, reflecting the need to supply precursors and energy to rapidly growing cells. Inactivation of the von Hippel-Lindau (VHL) gene, observed in the majority of ccRCCs, is one of the drivers of metabolic reprogramming [1]. Even in the presence of adequate oxygen levels, cancer cells shift away from the tricarboxylic acid cycle and oxidative phosphorylation toward increased aerobic glycolysis (Warburg effect). Increased synthesis of nucleotides, lipids, and amino acids is also a characteristic adaptation of cancer and ccRCC [2]. Lipid accumulation in ccRCC is associated with increased lipid synthesis and decreased beta-oxidation [3]. The accumulation of lipid droplets is a glutamine-dependent feature that distinguishes ccRCC from other tumors [4,5].

TMetastasis is the primary cause of cancer specific mortality in ccRCC. Numerous studies have correlated pathologic staging and grading [6] of ccRCC with multiomic features [7]. Grade and stage of the primary tumor are nevertheless surrogate markers, and are most closely associated with outcomes for low- or high-grade tumors,However, Identifying prognostic markers for ccRCC of intermediate grade and stage is more challenging. 10–20% of ccRCC patients present with synchronous metastases at initial diagnosis and 20–30% of the remaining patients develop metastasis during postoperative surveillance over a period of several years [8]. This study was designed to determine if metabolomic analysis of primary tumors could identify potential makers of risk for metastasis. Identifying prognostic biomarkers could create a diagnostic tool to aid clinicians in the management of patients with ccRCC.

ccRCC is characterized by reprogramming of energy metabolism [9]. Metabolomic profiles of plasma, urine and tissue have been published [[10–13]. Metabolomics is a powerful tool that comprehensively profiles small-molecule metabolites in biological systems; however, low metabolite coverage is a bottleneck in the field. High-performance chemical isotope labeling (CIL) LC-MS is a chemical derivatization approach that is combined with conventional LC-MS to separate metabolites based on their physiochemical properties. CIL LC-MS enhances detection by improving the separation and ionization efficiencies of the metabolites. The metabolome is divided into chemical group-based submetabolomes: carboxyl, amine–phenol, hydroxyl, and carbonyl. These cover up to 96% of the chemical entries in metabolome databases [14]. In this study, three submetabolomes were analyzed, including carboxyl, amine-phenol and hydroxyl, to achieve a high metabolomic coverage.

## Materials and methods

### Study design and study population

This was a retrospective case-control study of primary human tumors of ccRCC that compared metabolomic profiles of tumors that metastasized during a minimum follow-up period of 3 years (cases) versus those that did not (non-metastatic). Consented subjects who underwent surgical resection with intent to cure ccRCC were included. Continued inclusion required the availability of clinical notes, vital status, and imaging results. No effort was made to alter the number of males or females in the cohort. Subject BMI and weights were not recorded. Primary tumors from 222 subject samples were analyzed. 68 primary tumors were from subjects whose ccRCC subsequently metastasized at 3 years follow up. 3 subject tumors from the Canadian group originally classified as non-metastasis were ultimately staged as M1. Non-metastasis samples were from subjects who did not metastasize at 3 years follow up.. Subjects below age 18 and above 90 were excluded. An experienced urologic oncologist (MW) reviewed clinical records to determine the presence or absence of metastasis. The urological practice retains a high proportion of patients in follow-up. Imaging and other data were used to identify bone, non-regional lymph node, or visceral (lung or other internal organ) metastases. This is distinguished from recurrence in the original surgical field, whether following a partial or radical nephrectomy.. All specimens received a study accession number and metabolomics assays were conducted in a blinded fashion. All available clinical data elements are included as supporting information (S1 and S2 Tables).

### Sampling tumor tissue

Freshly obtained ccRCC primary tumor samples were collected from partial or complete nephrectomy specimens surgically removed with intent to cure. Samples were obtained from three clinical sites: Macon and Joan Brock Virginia Health Sciences at Old Dominion University, formerly Eastern Virginia Medical School UT Health San Antonio Mays Cancer Center Biorepository (Formerly University of Texas Health Science Center San Antonio), and the Alberta Cancer Research BioBank. Coded specimens and de-identified clinical data elements were provided for this study so that authors had no access to subject identifiers.

Visual examination of a frozen specimen (e.g., “gross examination”) typically allowed identification of the border between tumor and normal appearing kidney tissue. Each tissue specimen was sampled with a razor blade or cored with an 18-guage needle to obtain at least one 5 mg sample per tumor using a chilled surface to avoid thawing. The sample was extracted using 80% methanol for 24 hrs at 22^o^C. As described previously, immersion of tissue in aqueous methanol retains architecture while extracting metabolites [15] Tissue was removed from the aqueous methanol, placed in formalin, processed for histology using routine methods, and stained with hematoxylin and eosin (H&E). The histological sections of the remaining tissue cores were evaluated by a urologic pathologist (DAT). For consistency, we obtained normal samples that were procured only from the renal cortex. This aligns with the origin of ccRCC in the proximal convoluted tubular epithelial cells.

### Tissue banking and institutional review board approval

Coded specimens and de-identified clinical data elements were provided from three biorepositories for this study so that authors had no access to subject identifiers. Day, month, and year when data were accessed for research purposes were as follows. Alberta Cancer Research Biobank (https://www.acrb.ca/) 04/03/2020. Old Dominion University Biorepository and Histology 02/07/2020. UT Health San Antonio Mays Cancer Center Biorepository (Formerly University of Texas Health Science Center San Antonio) 09/06/2019. The research was conducted under IRB Protocol 17–08-NH-0168, Old Dominion University, formerly Eastern Virginia Medical School. Fresh tissues were snap frozen and stored at -80^o^ C.

### Materials

The metabolome quantification kit (NMT-6001-KT), chemical labeling kits NMT-4101-KT (dansyl-labeling kit for amine and phenol metabolomics), NMT-4167-KT (dansyl-labeling kit for carboxyl metabolomics), and NMT-4145-KT (dansyl-labeling kit for hydroxyl metabolomics) were obtained from Nova MT Inc., Nova Medical Testing, B108, 2011 94 Street NW, Edmonton, Alberta T6N11, Candad, ddfH1, Canada (Novamt.com). MS-grade water, acetonitrile (ACN) containing 0.1% formic acid, and water containing 0.1% formic acid were also obtained from Sigma-Aldrich. ACN and formic acid, used as elution buffers for LC-UV, were purchased from J.T. Baker-Avantor (Easton, PA, USA) and Sigma-Aldrich, respectively. Ultrapure water was obtained using a Milli-Q system (Merck Millipore, Darmstadt, Germany).

### Liquid chromatography/mass spectroscopy

#### Sample preparation.

Three chemical labeling methods were employed: amine–phenol, carboxyl, and hydroxyl. Before labeling, the total concentrations of the samples were determined using the NovaMT sample normalization kit. According to the quantification results, different sample volumes were taken to adjust the samples to the same concentration.

For samples with total concentrations smaller than 0.1 mM, the calculated volume of samples, or if not sufficient, all the available samples were utilized, completely dried, and re-dissolved in water. After centrifugation, the re-dissolved samples were split into four aliquots, including 3 for 3-channel labeling and one for backup. For the samples having total concentration between 0.1 mM to 0.17 mM, the calculated volume of samples was taken, completely dried and re-dissolved in water. After centrifugation, the re-dissolved samples were split into 5 aliquots, including 3 for 3-channel labeling, one for backup, and one for generation of the pooled sample. For samples with total concentrations higher than 0.17 mM, the calculated volume of samples was taken, completely dried, and re-dissolved in water. After centrifugation, the re-dissolved samples were split into 5 aliquots, including 3 for 3-channel labeling, one for backup, and one for the generation of a pooled sample with a larger volume.

#### Chemical labeling.

Dansylation and DmPA labeling were performed as described previously [16,17]. For amine/phenol labeling, 19 μL of water was added to the sample aliquot for analysis. The labeling protocol strictly followed the SOP provided in the kit. Briefly, 12.5 μL of buffer reagent A (Reagent A) and 37.5 μL of ^12^C2-labeling (for the individual samples and the pooled sample) or ^13^C2-labeling (for the pooled sample) reagent (Reagent B) were added to the samples. The samples were then vortexed and centrifuged. The mixtures were incubated at 40 °C for 45 min. 7.5 μL of quenching reagent (Reagent C) was then added to quench excess labeling reagent. The mixtures were incubated at 40 °C for another 10 min. Finally, 30 μL of the pH-adjusting reagent (Reagent D) was added.

For carboxyl channel labeling, 19 μL of ACN was added to the sample aliquot for analysis. The labeling protocol strictly followed the SOP provided in the kit. Briefly, 10 μL of the catalyzing Reagent A (Reagent A) and 25 μL of ^12^C2-labeling (for the individual and pooled samples) or ^13^C2-labeling (for the pooled sample) reagent (Reagent B) were added to the samples. The samples were then vortexed and centrifuged. The mixtures were incubated at 80°C for 60 min. Subsequently, 40 μL of quenching reagent (Reagent C) was added to quench excess labeling reagent. The mixtures were incubated at 80°C for another 30 min, and the chemical isotope labeling procedure was complete.

For hydroxyl channel labeling, 19 μL of ACN was added to the sample aliquot for analysis. The labeling protocol strictly followed the SOP provided in the kit. Briefly, 30 μL of reaction-activating Reagent A and 40 μL of ^12^C2-labeling (for the individual and pooled samples) or ^13^C2-labeling (for the pooled sample) reagent (Reagent B) were added to the samples. The samples were then vortexed and centrifuged. The mixtures were incubated at 60°C for 60 min. Subsequently, 5 μL of quenching reagent (Reagent C) was added to quench excess labeling reagent. The mixtures were incubated at 60 °C for another 10 min. Finally, 25 μL of the pH-adjusting reagent (Reagent D) was added.

#### Mixing to prepare study specific universal metabolome standard.

The ^12^C2-labeled individual sample was mixed with ^13^C2-labeled reference sample in equal volume. The mixture was prepared for LC-MS analysis. Prior to LC-MS analysis of the entire sample set, a quality control (QC) sample was prepared by mixing equal amounts of pooled ^12^C-labeled and ^13^C-labeled samples.

#### LC-MS analysis conditions.

LC-MS analysis was performed using the Rapid LC-MS Analysis for HP-CIL Metabolomics Platform, briefly described as follows: QC samples were injected every 20 samples to monitor instrument performance. A Thermo Scientific Vanquish LC equipped with an Agilent eclipse plus reversed-phase C18 column (150 x 2.1 mm, 1.8 μm particle size) linked to a Bruker Impact II QTOF Mass Spectrometer was used. The following conditions were used: *Mobil Phase A:* 0.1% (v/v) formic acid in water. *Mobil Phase B*: 0.1% (v/v) formic acid in acetonitrile. *Gradient*: t = 0 min, 25% B; t = 10 min, 99% B; t = 15 min, 99% B; t = 15.1 min, 25% B; t = 18 min, 25% B. *Flow Rate*: 400 μL/min. *Column Oven Temperature:* 40 °C. *Mass Range***:** m/z 220–1000

#### Data processing and cleansing.

The LC-MS data (including 14 QC samples) were first exported as. csv files using Bruker DataAnalysis 4.4. The exported data were uploaded to IsoMS Pro 1.2.8. Data quality checks and processing were conducted. The parameters used for data processing are as follows. Minimum m/z: 220. Maximum m/z: 1000. Saturation intensity: 20,000,000. Retention time tolerance: 9 s mass tolerance: 10 ppm.

Peak pairs without data present in at least 50.0% of samples in any group were filtered out. For example, for the amine–phenol submetabolome, an average of 1671 ± 185 peak pairs per run was detected. Peak pairs without data present in at least 50.0% of samples in any group were filtered out. After filtering, 1579 ± 150 peak pairs per run were retained. The data were normalized by the Ratio of Total Useful Signal.

### Metabolite identification

The parameters used for metabolite peak identification are as follows. Retention time tolerance for CIL Library ID: 30 s Retention time tolerance for the LI library ID A Channel: 205 s mass tolerance for the CIL library ID, LI library ID, and mass-based database ID: 10 ppm.

A three-tier approach was used to identify peak pairs. For tier 1 identification based on accurate mass and retention times, peak pairs were searched against the labeled metabolite CIL Library, which contained more than 141 dansyl (hydroxyl), 711 dansyl (amine & phenol), and 271 DmPA (carboxylic acid) experimental entries, including metabolites and dipeptides.

For tier 2 identification, peak pairs were searched against the linked identity library (LI Library), including over 7000 pathway-related metabolites, providing high-confidence putative identification results based on accurate mass and predicted retention time matches.

For tier 3 identification, the remaining peak pairs were searched, based on accurate mass match, against the MyCompoundID (MCID) library composed of 8,021 known human endogenous metabolites (zero-reaction library) and their predicted metabolic products from one metabolic reaction (375,809 compounds) (one-reaction library).

### Statistical analysis

A total of 222 separate subject tumors were analyzed. Fifty tumor-free kidney cortex tissue samples were also profiled. Three labeling methods were used as previously described. MetaboAnalyst [18] was used to compare all tumors with normal tissues. Supervised machine learning, using both linear and nonlinear Support Vector Machines (SVMs, analogous to Al-Rohil et al. [19]), was implemented with scikit-learn to construct classification models that distinguish between cases and non-metastatic tumors. Nonlinear SVMs utilize a Radial Basis Function (RBF) kernel. Furthermore, for the supervised machine learning, an additional dataset was created, combining features across all the different labeling methods per sample (referred to as the ‘Combined’ model). The goal of this combined dataset was to see if a combination of multiple chemistries can provide improved classification. Repeated cross-validation was employed to avoid overfitting and to provide a representative assessment of the classification performance of the model. The models demonstrated good classification performance, with an area under the Precision-Recall Curve (AUPRC) of 0.92 for the best-performing model.

To assess whether performance differences between linear and nonlinear SVM models were statistically significant, the full nested cross-validation procedure was repeated 100 times per dataset, each repetition using a different random fold partition. This yielded 100 paired (linear, nonlinear) performance estimates per dataset. The Wilcoxon signed-rank test (two-sided) was applied to the 100 paired differences (nonlinear minus linear) for each metric and dataset.

### Feature selection

To identify the most discriminating metabolic features between CASE and control samples, we employed a stability-based feature selection approach using L1-penalised linear support vector machines. The L1 penalty induces sparsity in the model coefficients, effectively setting the weights of non-informative features to zero and thereby performing embedded feature selection.

For each derivatization chemistry (amine-phenol, carboxyl, and hydroxyl) as well as the combined dataset, we performed 500 independent classification runs using 5-fold stratified cross-validation repeated 100 times, with a fixed regularization parameter C = 0.1. In each run, the indices of features with non-zero coefficients were recorded. Features were then ranked by their selection frequency, defined as the number of runs (out of 500) in which each feature received a non-zero weight. This repeated subsampling approach assesses the stability of feature selection and inherently addresses multiple testing concerns: features that are consistently selected across many independent data partitions are more likely to represent genuine biological discriminators rather than artefacts of a particular train/test split.

Metabolite Set Enrichment Analysis (MSEA) was performed using the Enhancement Analysis module and the Quantitative Enrichment Analysis mode in MetaboAnalyst. The algorithm was based on a global test for groups of genes [20]. The SMPDB option, referencing 99 datasets based on normal human metabolic pathways, was selected [21].

**Resources**

**Table:** 

Reagent or Resource	Source	Identifier
MetaboAnalyst	Wishart Lab	RRID:SCR_015539 v. 5.0
Human kidney tissue	Alberta Cancer Res biobank	https://www.acrb.ca/
Human kidney tissue	Mays Cancer Center Biorepository	https://uthscsa.edu/research/facilities-resources/biobank
Human kidney tissue	EVMS/ODU Biorepository	https://www.evms.edu/education/schools_institutes_centers_departments/pathology_anatomy/biorepository__histology/
Chemical labeling reagents	Nova Medical Testing	http://www.novamt.com
SVM and Statistical Support	Aspect Analytics	https://www.aspect-analytics.com/
tier 1 and 2 metabolite identification	Nova Medical Testing, Inc.	IsoMS Pro CIL LC-MS Metabolomics, NMT-5101-SW
tier 3 metabolite identification	MyCompound ID	https://www.mycompoundid.org/mycompoundid_IsoMS/

## Results

Our study population had an average age of disease onset (61 years) which was identical to that observed in the National Cancer Database [22]. The minimum age was 30 years, and the maximum age was 89 years at the time of surgery, with a male/female ratio of 1.97. The Hispanic representation was lower than expected (3.6%). African Americans (AA) were well represented in the control group (16.2%) but not in the case group (4.2%). The follow-up time for the AA group was not different from that of the other groups. All available clinical data elements for tumor stage and grade, type of surgical procedure, postoperative treatment, renal function, and relevant comorbities are included as supporting information in S1 and S2 Tables.

### Positively or putatively identified peak pairs

A total of 272 samples were analyzed (222 tumors and 50 normal samples). Out of the 3864 unique dansyl (hydroxyl) peak pairs detected, 3210 pairs (83.1%) could be positively identified or putatively matched. Among these, 375 peak pairs were identified as high-confidence results (tiers 1 and 2). Of the 1644 unique dansyl (amine & phenol) peak pairs detected, 1461 (88.9%) could be positively identified or putatively matched. Among these, 300 peak pairs were identified as high-confidence results (tiers 1 and 2). Of the 1913 unique carboxylic acid (DmPA) peak pairs detected, 1565 (81.8%) could be positively identified or putatively matched. Among them, 256 peak pairs were identified as high-confidence results (tier 1 and tier 2), which can be used for other analyses (e.g., pathway analysis). The results are summarized in Table 1.

**Table 1:** Metabolite identification for the three labeling methods.

Tier	Dansyl (Hydroxyl)	Dansyl (Amine & Phenol)	DmPA (Carboxylic Acid)
1 Positively Identified	16	147	78
2 Putative	359	153	178
3 zero reaction	577	377	411
3 one reaction	1748	566	651
3 two-reaction	510	218	247

### Normal tissue vs tumor tissue

Here tumors were compared with normal tissues. Metabolite Set Enrichment Analysis MESA to summarize tumor-related effects for the three chemistries are shown in Figs 1–3. A total of 222 tumor samples were compared with 50 non-tumor samples. MESA identifies biological pathways that are enriched by metabolites that are altered in tumors vs. normal tissues. Box plots showing representative tumor related metabolites are shown in Fig 4. Extracted ion chromatograms (EIC) of selected metabolites from a representative individual sample are provided in Supplemental S1 Fig as examples.

**Fig 1:**
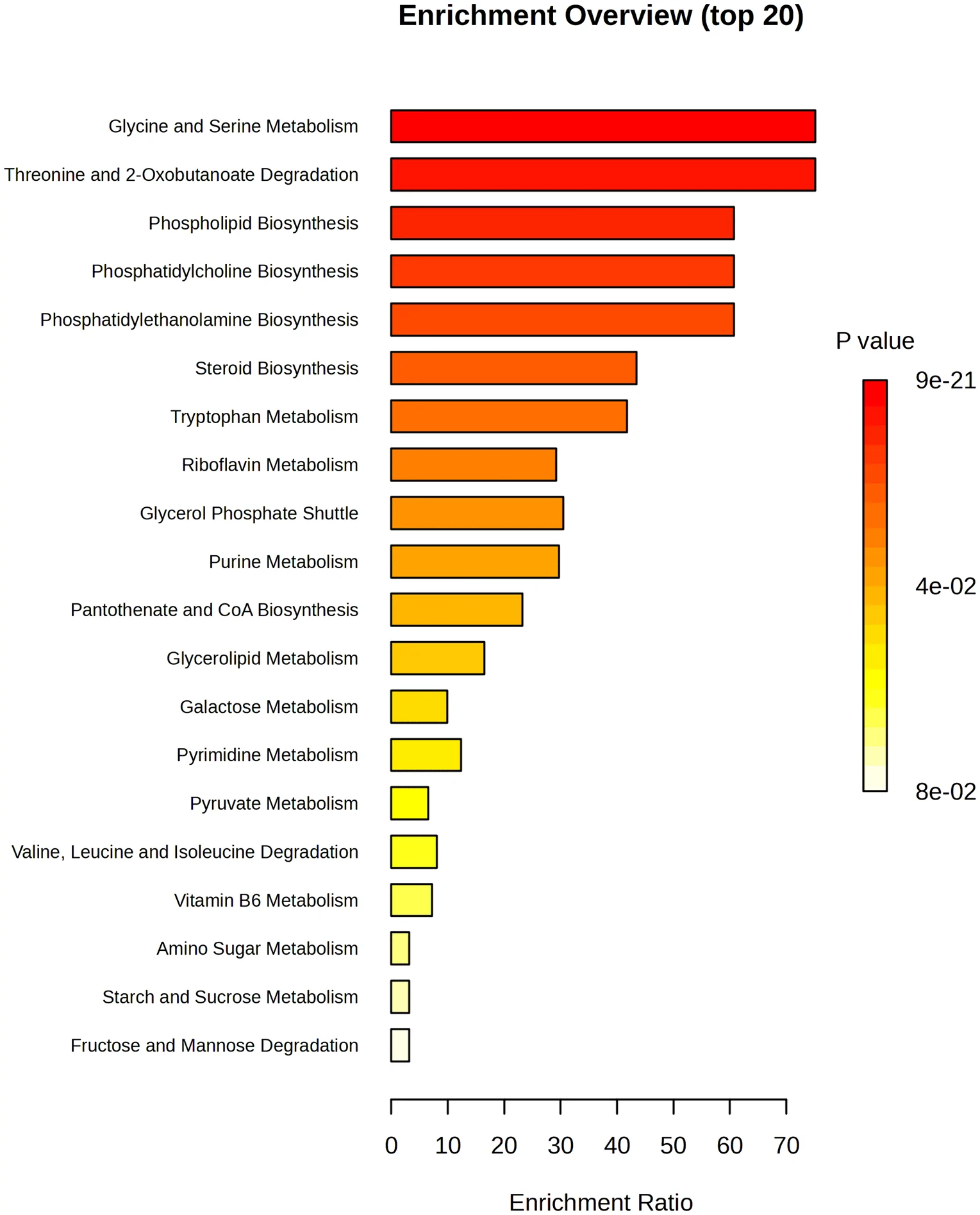
Metabolite Set Enrichment Analysis using Dansyl (Hydroxyl) chemical labeling. When significantly altered metabolites are overrepresented in a given pathway, the extent of the enrichment is displayed as the length of the bar. This is a calculated value compared to what is expected by random chance.

**Fig 2:**
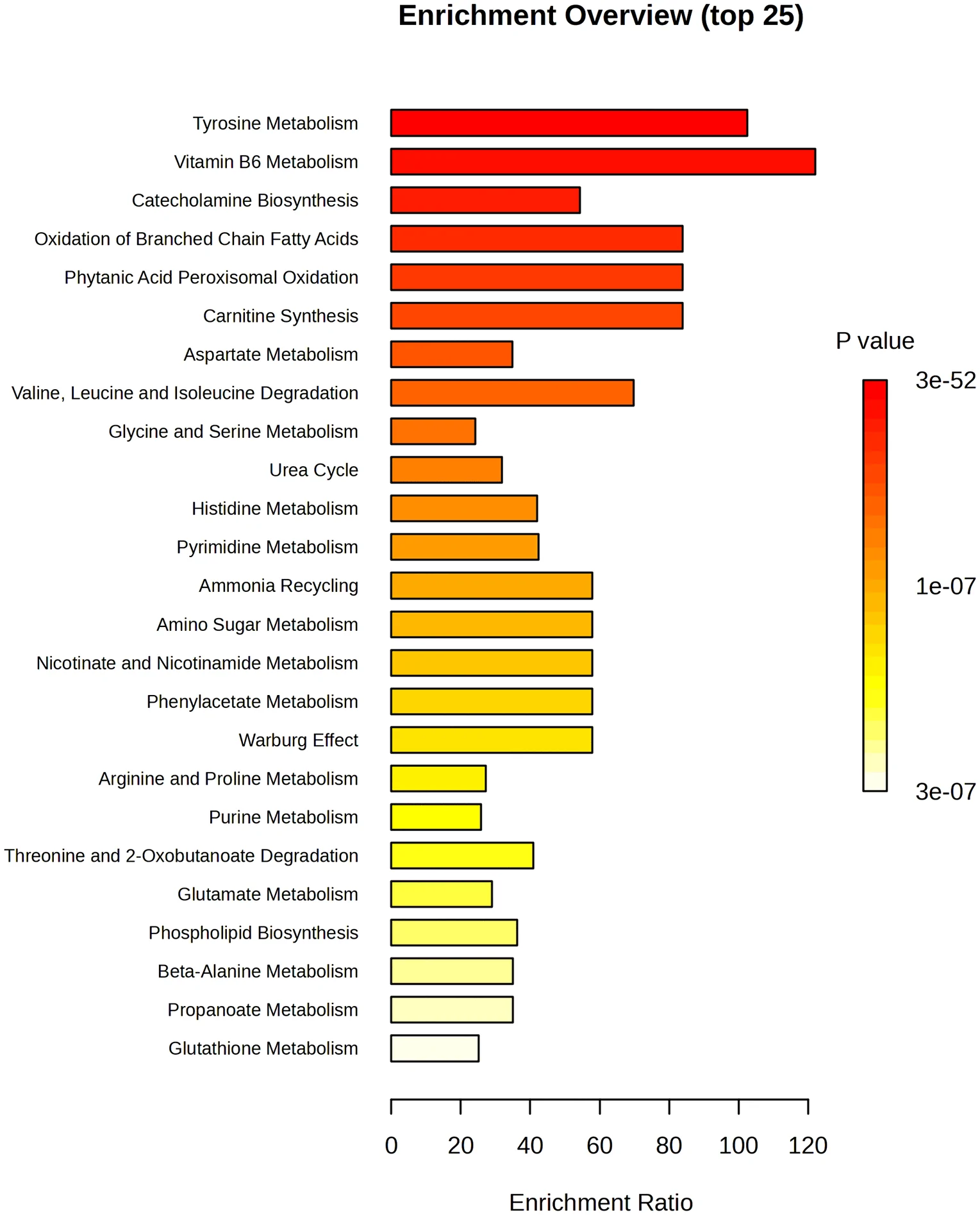
Metabolite Set Enrichment Analysis using Dansyl (Amine & Phenol) chemical labeling. See Fig 1 legend.

**Fig 3:**
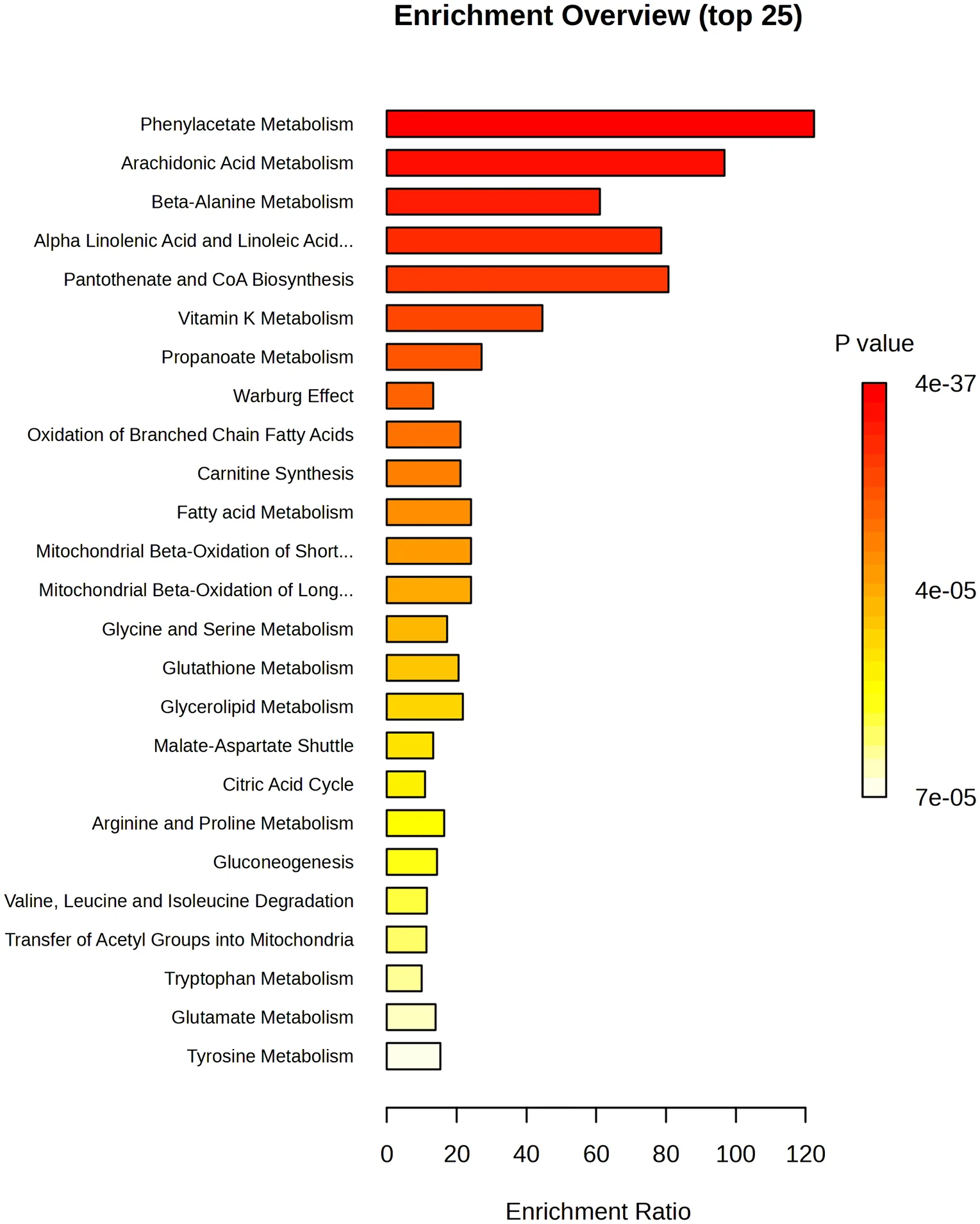
Metabolite Set Enrichment Analysis using DmPA (Carboxyl) chemical labeling. See Fig 1 legend.

**Fig 4:**
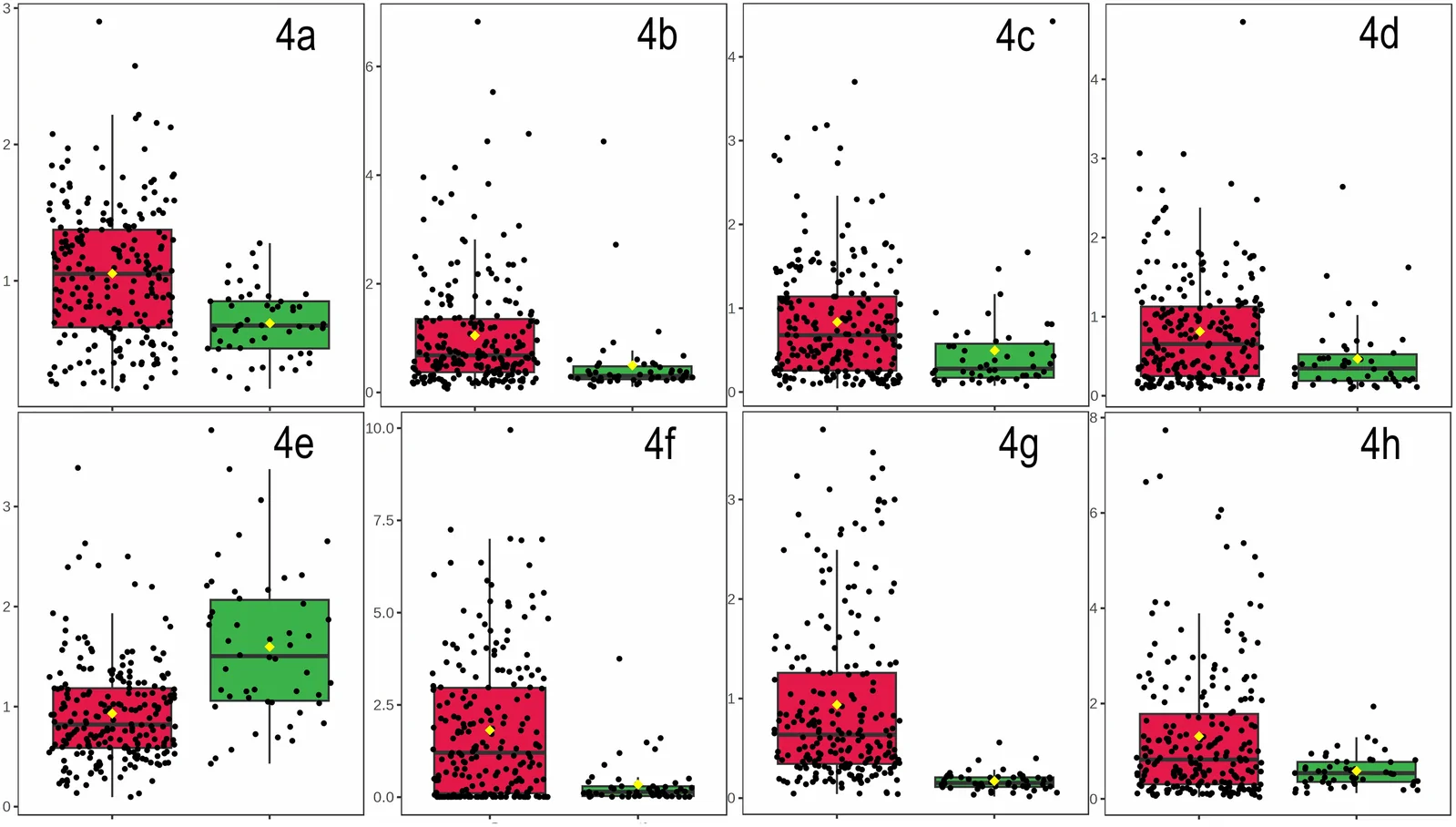
Box plots of frequently altered metabolites in ccRCC. Box plots show ^12^C/^13^C ratios for lactate (a),malate (b), citrate (c), cis-aconitate (d), succinate (e), glutathione disulfide (f), kyneurenine (g), and glutamyl-glutamic acid (h)., p < .05 for all metabolites. Green shows non-tumor tissue and red shows tumor tissue.

Box plots for selected metabolites frequently altered in ccRCC tumors are shown in Figs 4a-h). Lactate and malate, metabolic precursors of pyruvate, were increased in tumors compared to normal tissues, and the levels of the tricarboxylic acid cycle intermediates citrate and cis-aconitate were significantly increased. Glutamyl-glutamic acid, glutathione disulfide, and kynurenine levels were also higher in tumors than in non-tumor tissue. Succinate levels decreased.

### Tumors that metastasized vs Tumors that did not metastasize

A total of 154 non-metastatic tumors were compared with 68 tumors that had metastasized at 3 years. Definitively identified metabolites that statistically differentiated tumors that metastasized from those that did not include: 4-Hydroxybutanoic acid, 3-Aminosalicylic acid, indoxyl sulfate, norcotinine, 3,4- dihydroxyphenylpropanoic acid, glutamyl-glutamic acid, and N-ethylglycine. Both the linear and non-linear SVM models delivered good classification results across different chemistries, with the non-linear SVM models outperforming the linear models.

Table 2 summarizes the classification performance of the tuned linear and non-linear SVM models for each chemistry parameter. The ‘Combined’ row shows results of the combined data across all chemistries. Non-linear SVMs offer enhanced flexibility by allowing curved decision boundaries, enabling them to capture more complex patterns in the data. While this added complexity results in a higher number of model parameters, it also results in superior performance.

**Table 2:** Results of linear and non-linear SVM classifiers for the different chemistries.

	Features #	AU ROC Linear tuned	AUC PR Linear tuned	AU ROC Nonlinear tuned	AU PR Nonlinear tuned
amine-phenol	1644	0.78	0.88	0.81	0.90
carboxyl	1913	0.74	0.81	0.74	0.88
hydroxyl	3884	0.79	0.89	0.83	0.91
combined	7432	0.76	0.87	0.82	0.92

The SVM model parameters were tuned using Optunity Python library [23,24]. We employed nested 5-fold cross-validation with 100 iterations to ensure robust and reliable evaluation. This approach involved repeated tuning and training of the models using 5-fold cross-validation, iterating 100 times. The results in Table 2 reflect the average performance across iterations.

Non-linear modeling improved the AU Precision-Recall (PR) values to 0.9 or higher, for all chemistries. except the carboxyl chemistry (0.88). The AU PR column reports the total area under the PR curve, which plots the Precision against Recall (sensitivity). Precision measures the proportion of correctly labeled CASE samples out of all samples labeled as CASE, whereas recall (or sensitivity) measures the proportion of CASE samples correctly identified out of all CASE samples in the dataset. Table 3 summarizes the mean sensitivity of the non-linear SVM classifiers when the mean precision is constrained to a minimum of 80%, demonstrating the good sensitivity of the model at this precision threshold.

**Table 3:** Sensitivity and mean precision for the non-linear SVM classifiers. Sensitivity and precision are shown for the three chemistries, when setting a minimum precision of 80%.

	Mean Sensitivity	Mean Precision
amine-phenol	0.87	0.81
carboxyl	0.90	0.81
hydroxyl	0.92	0.81
combined	0.95	0.81

To determine whether the observed performance differences between linear and nonlinear SVM models were statistically significant, the nested cross-validation procedure was repeated 100 times per dataset, each with a different random fold partition. Table 4 summarizes the results of this comparison. The nonlinear SVM significantly outperforms the linear SVM across all dataset (Wilcoxon signed-rank test, two-sided).

**Table 4:** SVM classification performance. Statistical comparison of linear vs nonlinear SVM classification performance across 100 repeated nested cross-validation iterations. Values reported as mean ± standard deviation. p-values from two-sided Wilcoxon signed-rank test.

Dataset	Linear AU ROC	Linear AU PR	Nonlinear AU ROC	Nonlinear AU PR	p (ROC)	p (PR)
Amine-Phenol	0.761 ± 0.021	0.870 ± 0.016	0.806 ± 0.015	0.890 ± 0.012	8.5e-18	5.6e-14
Carboxyl	0.706 ± 0.022	0.835 ± 0.018	0.748 ± 0.015	0.863 ± 0.012	2.4e-17	1.5e-16
Hydroxyl	0.761 ± 0.021	0.873 ± 0.014	0.802 ± 0.014	0.900 ± 0.009	2.0e-17	1.7e-17
Combined	0.751 ± 0.022	0.868 ± 0.014	0.820 ± 0.015	0.908 ± 0.010	4.0e-18	4.5e-18

### Feature selection

Using the stability-based feature selection approach described in the Methods section, on average each individual model retained approximately 90–103 features with non-zero coefficients out of the full feature space (89.4 ± 5.7 for Amine-Phenol, 91.5 ± 5.4 for Carboxyl, 94.8 ± 6.2 for Hydroxyl, and 103.3 ± 6.5 for the combined dataset). The 10 most frequently selected features per chemistry across the 500 runs were retained for further biological interpretation. These top-ranking features showed very high selection stability, appearing on average in 458–475 out of 500 runs across the four datasets (470.4 ± 23.9 for Amine-Phenol, 472.5 ± 20.1 for Carboxyl, 458.2 ± 23.4 for Hydroxyl, and 475.2 ± 17.7 for the combined dataset). Selected features were cross-referenced against compound databases by matching neutral mass and normalized retention time at three identification confidence levels: tier 1 (positive identification from the CIL library), tier 2 (high-confidence putative identification from the LI library), and tier 3 (putative identification from the MyCompoundID library).

This search yielded the following metabolites with tier 1 or 2 idendification, that differentiated tumors that metastasized from those that did not: 4-Hydroxybutanoic acid, 3-Aminosalicylic acid, norcotinine, 3,4- dihydroxyphenylpropanoic acid, glutamyl-glutamic acid, and N-ethylglycine. tier 3 identification: Indoxyl sulfate.

## Discussion

ccRCC is characterized by reprogramming of energy metabolism [25–29]. Metabolic changes in ccRCC include increased glucose uptake and utilization [30], e.g., the Warburg effect. Metabolomic profiling of ccRCC tumors show that the Warburg effect is grade-dependent with upregulation of aerobic glycolysis and lactate fermentation in high grade vs low grade tumors [31]. Comparative analysis of metabolite and gene expression data confirmed that additional pathways related to HIF-alpha, degradation of methionine and choline, and acetyl-CoA biosynthesis are observed in ccRCC [32]. In addition, glucose is shunted toward the pentose phosphate shunt pathway (PPP) in ccRCC [33]. A study combining metabolomic and transcriptomic data identified a role for MUC1 as a metabolic regulator and suggesting that CA15−3, the soluble form of MUC1, may serve as a prognostic marker for more aggressive ccRCC [34]. Further studies suggest that metabolomic reprogramming may play a role in responsiveness to anti-neoplastic drugs [35]. These and other studies support a role for continued metabolomic profiling of ccRCC to identify prognostic and potential theraonostic markers.

Our main goal was to identify prognostic metabolite markers for metastasis in primary ccRCC tumors. Our approach to metabolite analysis leveraged the strengths of non-radioactive chemical labeling and the use of a study-specific standard for normalization. The output was a ^12^C/^13^C ratio that reflected the steady-state concentration of metabolites in the tissues examined. Increased and decreased steady-state concentrations reflect the production and consumption of enzymatic substrates and products, respectively. However, the lack of change in steady-state concentrations cannot always be interpreted as a lack of involvement in altered metabolic fluxes [36].

We first compared the metabolic profiles of tissues from all available ccRCC tumors with those of non-tumor normal kidney tissues. Increased aerobic glycolysis, with increased flux through pyruvate, is a characteristic of metabolic reprogramming in cancer. In ccRCC [37], HIF-1α also promotes lactate production and decreases pyruvate entry into mitochondria. In our samples, the levels of lactate and malate, which are both metabolic precursors of pyruvate, were higher in tumors than in normal tissues. The levels of the tricarboxylic acid cycle intermediates citrate, cis-aconitate, and malate were also significantly increased in tumors. Succinate levels decreased in the tumors. The tryptophan metabolism pathways were also altered. Tryptophan is used for protein synthesis, serotonin synthesis, and kynurenine [38,39].

Metabolites altered in primary tumors that metastasized versus those that did not are discussed as follows. 4-Hydroxybutanoic acid is a precursor and metabolite of gamma-aminobutyric acid (GABA) found in CNS, wine, beef, and citrus. *In vitro* studies have suggested that GABA may influence the invasiveness of renal cell carcinomas [40]. A recent study suggested that GABA metabolism may play a role in overcoming sunitinib resistance [41]. No previous publications on this as a biomarker for renal cell carcinoma are currently available.

Glutamyl-glutamic acid is elevated in ccRCC and is a precursor of Glutathione. Glutamyl-glutamic acid is produced by the combination of the gamma-carboxyl group of glutamic acid and the amino group of another glutamic acid molecule. Glutamyl-glutamic acid is linked to fatty acid synthesis through glutamine/glutamate-driven anaplerosis and reductive carboxylation [42], which provides the acetyl-CoA required for lipid synthesis [43]. Generally, ccRCC tumors have increased levels of gamma-glutamyltransferase 1 (GGT1) and gamma-glutamylglutamic acid [44]. Functional transcripts of GGTs are classified as type I, and are membrane-bound enzymes found mainly in the liver [26]. Glutamine is essential for fatty acid synthesis, and the accumulation of lipid droplets is dependent on glutamine^4^ in renal cell carcinoma.

The indolic metabolic pathway produces indoxyl sulfate, which acts as a uremic toxin in patients with chronic renal failure. It can therefore be influenced by renal function. It is produced stepwise by tryptophanase-expressing gastrointestinal bacteria from dietary tryptophan in the large intestine and is increased in ccRCC tissues [45]. Indole is an intermediate that is then converted to Indoxyl Sulfate by liver microsomal cytochrome p450 enzymes [46]. In patients with chronic renal failure, indoxyl sulfate exacerbates colorectal cancer by activating the aryl hydrocarbon receptor repressor (AhR) and phosphoinosidtide 3-kinase (PI3K) protein kinase B (Akt) pathways [47].

3,4-Dihydroxyphenylpropanoic acid is produced from caffeic acid by gut microbiota [48] and inhibits histone deacetylase activity [49]. It is found in coffee and apples, and may therefore be influenced by diet.

N-ethylglycine is a derivative of glycine, which is rarely endogenously generated in normal cells. N-ethylglycine in humans can originate from pharmacological and dietary/microbial sources. It is a metabolite of lidocaine that reduces pain by inhibiting glycine uptake via glycine transporter 1 (GlyT1) [50]. One study showed that it is elevated in cancer patients with bone metastases [51]. Although glycine utilization for single-carbon-based synthesis of nucleic acids is increased in ccRCC, N-ethylglycine is not metabolized by the usual glycine-related pathways. Increased flux through serine and glycine pathways is observed in cancers, including ccRCC, particularly in the activity of glycine decarboxylase (GLDC). Conflicting results have been reported, where GLDC is reduced in renal cell carcinoma cell lines and in human ccRCC tissue [52].

Norcotinine is a minor nicotine related metabolite [53]. Related to tobacco use, it is therefore not aligned with endogenous biomarkers.

3-aminosalicylic acid is an exogenous salicylic acid derivative and therefore not likely to be of clinical significance..

In summary, our approach to metabolomics combining chemical labeling and LC/MS is a promising method of biochemical analysis for identifying prognostic markers of outcomes such as RCC metastasis. Study strengths include procurement of samples from multiple sites, avoiding single site biases resulting from sample handling, processing and storage.

Future studies should include samples obtained from multiple centers to increase sample size. Future studies could also survey patient cohorts for up to 5 years rather than 3 years in order to approximate the clinical surveillance time per the NCCN guidelines.

## Supporting information

S1 Fig Extracted Ion Chromatograms. Extracted ion chromatograms (EIC) of selected metabolites from a representative invidvidual sample.
(TIF)

S1 Table Clinical Data Elements. Clinical data elements for gender, age at accrual, noadjuvant treatment (Neo Tx), necrosis, grade, creatinine, and BUN.
(DOCX)

S2 Table Clinical Data Elements. Clinical data elements for type of surgery, comorbidities, and follow-up treatment.
(DOCX)
