# Supplementary material for: Metabolomic profiling of renal cell carcinoma to identify prognostic markers of metastasis
Source: PLoS One. 2026 Nov 11;21(11):e0361404. doi: 10.1371/journal.pone.0361404 (PMC13754214; doi:10.1371/journal.pone.0361404)
Supplement: Supplementary file 1 [file pone.0361404.s002.docx]

| **PID** | **CTPS ID** | **Gender** | **Age at Accrual** | **Race** | **Met NonMet** | **Neo Tx** | **Stage** | **Necrosis** | **Grade** | **Creat umol/L (35-100)** | **BUN mmole/L (2.5-8.5)** |
| --- | --- | --- | --- | --- | --- | --- | --- | --- | --- | --- | --- |
|  | RT1012 | M | 71 | W | Met | no | T3a N0 M0 | N/A | 3 | 142 | 15.1 |
|  | RT1054 | M | 68 | W | Met | no | T3a Nx M0 | N/A | 2 | 151 | 11.1 |
|  | RT1165 | M | 56 | W | Met | no | T3b N0 M1 | N/A | 4 | 83 | 4.5 |
|  | RT1455 | M | 61 | W | NonMet | no | T3b N0 M1 | N/A | 2 | 77 | 12.8 |
|  | RT1499 | M | 51 | W | NonMet | no | T2 N0 M1 | N/A | 3 | 89 | 5.7 |
|  | RT1509 | M | 57 | W | Met | no | T1b N0 M0 | N/A | 2 | 104 | 6 |
|  | RT1584 | M | 80 | W | NonMet | no | T3b N0 M1 | N/A | 2 | 117 | 9.5 |
|  | RT1629 | N/A | N/A | N/A | NonMet | N/A | T2 N0 M0 | N/A | 2 | 110 | 8 |
|  | RT1727 | M | 46 | W | Met | no | T2 N0 M1 | Minimal | 2 | 125 | 7.9 |
|  | RT1828 | M | 46 | W | Met | no | T3b N0 M1 | Moderate | 3 | N/A | N/A |
|  | RT832 | F | 54 | Black | Met | no | T3b N0 M0 | Extensive | 3 | 77 | 3.8 |
|  | RT858 | M | 75 | W | NonMet | no | T4 N0 M1 | N/A | 2 | 135 | 9.8 |
|  | RT862 | M | 41 | W | Met | no | T2 N0 M0 | Minimal | 2 | 93 | 6 |
|  | UT458 | M | 76 | W | Met | no | not available | N/A | 2 | 135 | 9 |
|  | UT486 | N/A | N/A | N/A | Met | N/A | pT2pNxpMx | N/A | 3 | 128 | 6.9 |
|  | UT517 | M | 55 | W | Met | no | T3bNx | N/A | 3 | 1193 | 20.4 |
|  | UT576 | M | 58 | A | Met | no | pT1aN0M0 | N/A | 2 | 102 | 4.1 |
|  | UT583 | N/A | N/A | N/A | Met | N/A | pT3b N2 | N/A | 2 | 119 | 9.4 |
|  | UT609 | M | 36 | W | Met | no | pT2 | N/A | 3 | 109 | 6 |
|  | UT626 | M | 66 | W | NonMet | no kidney notes | not available | N/A | 2 | 147 | 9.2 |
|  | UT646 | M | 66 | W | NonMet | no | T3 | N/A | 2 | N/A | N/A |
|  | UT733 | M | 59 | W | Met | no | T3b N2 M0 | N/A | 3 | 86 | 6.8 |
|  | UT742 | M | 58 | A | NonMet | no early kidney notes | T1b N0 M0 | N/A | 2 | N/A | N/A |
|  | UT761 | M | 73 | W | NonMet | no | T3b N0 M0 | N/A | 2 | 70 | 9 |
|  | UT769 | M | 52 | W | Met | no | T2 N0 M0 | N/A | 2 | 80 | 7.5 |
|  | UT837 | N/A | N/A | N/A | NonMet | N/A | T1a N0 M0 | N/A | 2 | N/A | N/A |
|  | UT865 | M | 50 | W | Met | no | T2 N0 M0 | N/A | 1 | N/A | N/A |
|  | UT871 | M | 80 | W | Met | no | T3b N1 M1 | N/A | 3 | 98 | 6.7 |
|  | F10012 | F | 76 | W | NonMet | no | T3b N0 M0 | N/A | 2 | 69 | 4.5 |
|  | R105 | F | 60 | W | NonMet | no | T3b N0 M0 | N/A | 4 | 63 | 3.8 |
|  | R236 | F | 39 | W | NonMet | no | T1a N0 M0 | N/A | 3 | 136 | 5.7 |
|  | R238 | F | 83 | W | Met | no | T3a N0 M0 | N/A | 3 | 86 | 6.2 |
|  | R279 | F | 52 | W | Met | no | T1b N0 M0 | N/A | 3 | 93 | 6.8 |
|  | R420 | F | 46 | W | Met | no | T3b N0 M1 | N/A | 3 | 109 | 6.6 |
|  | R440 | F | 37 | W | NonMet | no | T1a N0 M0 | N/A | 1 | 66 | 4.7 |
|  | R487 | F | 42 | W | NonMet | no | T3b N0 M0 | N/A | 3 | 96 | 5 |
|  | R525 | F | 41 | W | NonMet | no | T3b N0 M0 | N/A | 9 | 142 | 7.4 |
|  | R56 | F | 53 | W | NonMet | no | T1a N0 M0 | N/A | 3 | 102 | 7.9 |
|  | R599 | F | 78 | W | NonMet | no | T1a N0 M0 | N/A | 3 | 123 | 7.3 |
|  | R85 | F | 45 | W | Met | no | T3b N0 M0 | N/A | 3 | 142 | 4.9 |
|  | R87 | F | 53 | W | NonMet | no | T3b N0 M0 | N/A | 3 | 100 | 4.9 |
|  | **GUTB** |  |  |  |  |  |  |  |  | **Creat mg/dl** | **BUN mg/dl** |
|  | 284-A | F | 62 | W | NonMet | N/A | pT1aN0MX | N/A | 2/4 | 0.9 | 13 |
|  | 294-A | M | 56 | W | NonMet | N/A | pT1bN0MX | N/A | 3/4 | 0.8 | 14 |
|  | 296-D | M | 67 | W | NonMet | N/A | pT1aN0MX | N/A | 3/4 | 1.1 | 11 |
|  | 297-A | M | 72 | N/A | NonMet | N/A | pT1aN0MX | N/A | 2/4 | 1.1 | 12 |
|  | 298-A | M | 52 | W | NonMet | N/A | pT1N0MX | N/A | 1/4 | 1.1 | 21 |
|  | 300-A.2 | F | 48 | W | NonMet | N/A | T3bN0MX | N/A | 1/4 | 0.7 | 15 |
|  | 301-A.2 | M | 30 | N/A | NonMet | N/A | T2aN0MX | N/A | 2-3/4 | 0.9 | 10 |
|  | 302-C.2 | M | N/A | N/A | NonMet | N/A | T1aN0MX | N/A | 1/4 | 0.9 | 13 |
|  | 304-E.A.2 | M | 68 | W | NonMet | N/A | T1aN0MX | N/A | 2/4 | 1.2 | 16 |
|  | 305-A.2 | F | 60 | W | NonMet | N/A | NA | N/A | 1/4 | 1.1 | 20 |
|  | 308-C.2 | M | 43 | W | NonMet | N/A | T2N0MX | N/A | 1/4 | 1.1 | 15 |
|  | 310-C.E.2 | M | 54 | N/A | NonMet | N/A | T1N0MX | N/A | 2/4 | 1.2 | 13 |
|  | 319-C | M | 60 | W | NonMet | N/A | T2aN0MX | N/A | 3/4 | 0.86 | 17 |
|  | 289-A | M | 58 | W | Met | N/A | pT1bN0MX | N/A | 4/4 | 2.1 | 34 |
|  | 292-F | M | 65 | W | Met | N/A |  | N/A | N/A | x | x |
|  | 295-F | M | 62 | W | Met | N/A | pT3aN1MX | N/A | 3/4 | 0.7 | 14 |
|  | 307-U.2 | F | 56 | N/A | Met | N/A | T3bN2MX | N/A | 3/4 | 0.6 | 9 |
| **PID** | **EVMS Bio** |  |  |  |  |  |  |  |  |  |  |
| 1 | BR101-48 | M | 76 | White | Met | No | T3b N0 M1 | N/A | III | 1.7 | 32 |
| 3 | BR101-42 | F | 72 | White | Met | Yes | T3a NX MX | N/A | II | 0.6 | 12 |
| 4 | 29-T (5) | F | 56 | Unknown | NonMet | No | T3b NX MX | N/A | IV | 0.9 | 20 |
| 9 | BR101-27-N | F | 78 | Black | NonMet | No | T1b NX MX |  |  | 0.9 | 17 |
| 9 | BR101-27-T |  |  |  | NonMet |  |  | Present | IV |  |  |
| 10 | BR101-49 | F | 51 | White | Met | No | T3a NX MX | N/A | II-III | 0.7 | 13 |
| 11 | BR107-69-N | M | 48 | White | NonMet | No | T1 NX MX |  |  | 1.1 | 15 |
| 11 | BR107-69-T |  |  |  | NonMet |  |  | N/A | II |  |  |
| 12 | BR112-119-T | M | 63 | White | NonMet | No | T1b NX MX | N/A | II |  |  |
| 12 | 119-N |  |  |  | NonMet |  |  |  | N/A |  |  |
| 13 | 3-T (6) | F | 59 | White | NonMet | No | T1b NX MX | N/A | II | 0.5 | 15 |
| 18 | BR101-2 | M | 56 | White | NonMet | No | T1a N0 MX | N/A | II | 19 | 19 |
| 19 | BR101-16-N | M | 58 | White | NonMet | No | T1a NX MX |  |  | 1 | 14 |
| 19 | BR101-16-T |  |  |  | NonMet |  |  | N/A | I/II |  |  |
| 20 | 120-N | M | 59 | White | NonMet | No | T1a NX MX | N/A | I-II | 1.2 | 27 |
| 21 | BR112-121-T |  | N/A | N/A | NonMet | No | T1a NX MX | N/A | II/IV | 1.8 | 24 |
| 21 | 121-N |  |  |  | NonMet |  |  | N/A | N/A |  |  |
| 22 | 61-N (5) | M | 61 | White | Met | No | T1b NX MX |  |  | 1.3 | 20 |
| 22 | 61-T (6) |  |  |  | Met |  |  | N/A | II/IV |  |  |
| 23 | BR101-28 | F | 56 | Unknown | NonMet | No | T3b NX MX | N/A | II | 1.6 | 24 |
| 24 | BR101-4 | M | 61 | White | NonMet | No | T1b NX MX | N/A | III | 0.9 | 15 |
| 29 | BR107-65 | M | 42 | White | NonMet | No | T2 NX MX | N/A | II | 0.8 | 11 |
| 30 | BR107-43 | M | 77 | White | Met | No | T3 NX MX | N/A | III | 0.6 | 13 |
| 31 | BR112-136-N | F | 62 | Black | NonMet | No | T2 NX MX |  |  | 1 | 12 |
| 31 | BR112-136-T |  |  |  | NonMet |  |  | N/A | II |  |  |
| 32 | 116-N | M | 91 | White | NonMet | No | T2 NX MX |  | II/IV | 0.9 | 25 |
| 33 | BR107-87-N | F | 67 | White | NonMet | No | T1 NX MX |  |  | 0.6 | 13 |
| 33 | BR107-87-T |  |  |  | NonMet |  |  | N/A | II |  |  |
| 34 | BR107-63-1T | F | 39 | Asian | NonMet | No | pT1a NX MX | N/A | III | 0.7 | 9 |
| 35 | BR107-67 | M | 45 | White | NonMet | No | T2 N0 MX | N/A | II | 0.7 | 12 |
| 36 | BR107-59-N |  | N/A | N/A | Met | No | T4 N0 MX |  |  | 1.5 | 13 |
| 36 | BR107-59-T |  |  |  | Met |  |  | N/A | IV |  |  |
| 37 | BR112-148-T | F | 58 | White | NonMet | No | T2 N0 MX | N/A | II/III | 0.6 | 12 |
| 38 | BR112-151-T | M | 63 | White | Met | No | T3a NX MX | N/A | II/IV | 0.9 | 10 |
| 39 | BR107-86 | M | 66 | White | NonMet | No | T1a NX MX | N/A | II/IV | 1.1 | 19 |
| 40 | BR107-89 |  | N/A |  | NonMet | No | T1a NX MX | N/A | II/IV | N/A | N/A |
| 41 | BR107-52-N | M | 62 | White | Met | No | T4 N1 M1 |  |  | 1.9 | 30 |
| 41 | BR107-52-T |  |  |  | Met |  |  | PRESENT | IV |  |  |
| 42 | BR112-177-1T | F | 80 | White | NonMet | No | T1b NX MX | N/A | III/IV | 0.9 | 16 |
| 43 | BR112-057-1T | F | 62 | White | Met | No | T3b N0 MX | PRESENT | IV | 0.6 | 15 |
| 46 | BR107-80-N | M | 64 | White | NonMet | No | T1 NX MX |  | N/A | 0.7 | 12 |
| 46 | BR107-80-T |  |  |  | NonMet |  |  | N/A |  |  |  |
| 48 | BR112-178-1T | F | 75 | White | Met | No | T3a NX MX | PRESENT | IV | 0.5 | 15 |
| 49 | BR112-186-1T |  |  |  | Met | No | T3b NX MX | N/A | II | 0.7 | 16 |
| 51 | BR112-164-1T | M | 65 | White | NonMet | No | T2 NX MX | PRESENT | III | 0.9 | 16 |
| 52 | BR112-145-T | M | 48 | Unknown | Met | No | T2 NX MX | N/A | II/IV | 0.9 | 20 |
| 53 | BR112-149-T | F | 60 | White | NonMet | No | T1a NX MX | N/A | II/IV | 1.3 | 14 |
| 55 | BR107-85-N | M | 66 | White | NonMet | No | T1b NX MX |  | II/IV | 1 | 3.9 |
| 55 | BR107-85-T |  |  |  | NonMet |  |  | N/A |  |  |  |
| 56 | BR107-91-N | F | 80 | White | NonMet | No | T2 NX MX |  |  | 2.2 | 36 |
| 56 | BR107-91-T |  |  |  | NonMet |  |  | PRESENT | II |  |  |
| 57 | BR107-81-N | F | 64 | White | NonMet | No | T1a NX MX |  | I | 0.9 | 14 |
| 57 | BR107-81-1T |  |  |  | NonMet |  |  | N/A |  |  |  |
| 58 | BR112-150-T | M | 65 | White | NonMet | No | T1a NX MX | N/A | II/IV | 1.2 | 13 |
| 59 | BR112-187-1T | M | 49 | White | NonMet | No | T1a NX MX | N/A | III/IV | 1 | 19 |
| 60 | BR112-147-T | M | 70 | White | Met | No | T3a NX MX | PRESENT | III | 1 | 21 |
| 62 | BR112-124-T | F | 49 | White | NonMet | No | T1a NX MX | PRESENT | II | 0.5 | 21 |
| 66 | BR112-125-T | M | 59 | White | Met | No | T1b N0 MX | N/A | II | 1.1 | 11 |
| 67 | BR107-78-N |  | N/A | N/A | NonMet | No | T1 NX MX |  | II | 1 | 16 |
| 67 | BR107-78-T |  |  |  | NonMet |  |  | N/A |  |  |  |
| 68 | BR107-76 | M | 57 | White | NonMet | No | T1a NX MX | N/A | III/IV | 0.8 | 17 |
| 70 | BR112-152-T | M | 48 | White | NonMet | No | T2 NX MX | N/A | II/IV | 0.8 | 12 |
| 71 | BR112-127-T(T2) | F | 61 | White | Met | No | T3a N0 M0 | PRESENT | II | 0.8 | 16 |
| 72 | BR112-128-T | M | 66 | White | NonMet | No | T2 NX MX | N/A | II | 1.6 | 31 |
| 72 | 128-N |  |  |  | NonMet |  |  |  |  |  |  |
| 73 | BR112-159-T | F | 68 | White | NonMet | No | T1a NX MX | N/A | III/IV |  |  |
| 74 | BR107-83 | M | 64 | White | NonMet | No | T1b NX MX | N/A | III-IV/IV | 0.9 | 18 |
| 75 | BR142-075-T | M | 55 | White | NonMet | No | T1b NX MX | N/A | II | 1 | 10 |
| 76 | BR107-73 | F | 50 | White | NonMet | No | T1a N0 MX | N/A | I/IV |  |  |
| 80 | BR112-165-1T |  | N/A | N/A | Met | No | T3 N0 M1 | N/A | III | 0.7 | 7 |
| 81 | 7-N (5) | M | 68 | White | NonMet | No | T1b NX MX |  | II | 1 | 23 |
| 81 | 7-T (6) |  |  |  | NonMet |  |  | N/A |  |  |  |
| 84 | BR112-166-1T | M | 39 | White | Met | No | T3b NX MX | PRESENT | III | 1 | 17 |
| 85 | BR112-167-1T |  |  |  | Met | No | T2 NX MX | N/A | IV/IV | 1.2 | 13 |
| 86 | BR107-79-N | F | 64 | White | NonMet | No | T1 NX MX |  | II | 0.8 | 16 |
| 86 | BR107-79-T |  |  |  | NonMet |  |  | N/A |  |  |  |
| 87 | BR112-098-T (T2) | M | 54 | White | NonMet | No | T1b NX MX | N/A | II | 0.8 | 11 |
| 89 | BR107-30-N | M | 48 | Black | NonMet | No | T1b NX MX |  | II/IV | 1 | 15 |
| 89 | BR107-30-T |  | N/A |  | NonMet |  |  | N/A |  |  |  |
| 91 | BR112-161-T |  | N/A | N/A | NonMet | No | T3a NX MX | N/A | IV/IV | 1.2 |  |
| 92 | BR107-053 (T3) | M | 58 | White | Met | No | T3a NX MX | N/A | II | 1.2 | 27 |
| 92 | BR107-53 |  |  |  | Met |  |  |  |  |  |  |
| 94 | BR107-005 | M | 63 | White | NonMet | No | T3a N0 MX | N/A | IV | 1.2 | 8 |
| 96 | BR112-126-1T | M | 65 | White | NonMet | No | T3a N0 MX | PRESENT | IV | 1.4 | 18 |
| 97 | BR112-169-2T | F | 72 | White | Met | No | T1b NX MX | PRESENT | III | 0.7 | 7 |
| 98 | BR107-71 | M | 49 | White | NonMet | No | T3a NX MX | N/A | III/IV | 0.8 | 9 |
| 99 | 14-N (2) | F | 66 | Black | NonMet | No | T1b NX MX |  | IV | 0.7 | 8 |
| 99 | 14-T (5) |  |  |  | NonMet |  |  | PRESENT |  |  |  |
| 100 | BR112-137-T | F | 63 | White | NonMet | No | T1b NX MX | N/A | III/IV | 0.6 | 20 |
| 103 | BR107-90 | M | 76 | Black | NonMet | No | T1a NX MX | N/A | III/IV | 1.1 | 27 |
| 109 | BR107-008-N | M | 43 | White | NonMet | No | T1b NX MX |  |  | 0.8 | 14 |
| 109 | BR107-008-T |  |  |  | NonMet |  |  | N/A | II/IV |  |  |
| 110 | BR112-141-T | M | 66 | Black | NonMet | No | T2 NX MX | N/A | II/IV | 1.32 | 27 |
| 111 | BR112-138-N | M | 33 | White | NonMet | No | T1b NX MX |  |  | 0.7 | 23 |
| 111 | BR112-138-T |  |  |  | NonMet |  |  | N/A | II |  |  |
| 112 | BR112-170-1T | M | 74 | White | NonMet | No | T1b NX MX | N/A | II | 1.9 | 43 |
| 114 | BR107-21 | M | 58 | White | NonMet | No | T2 N0 MX | N/A | III | 1.8 | 19 |
| 115 | BR112-139-N | M | 61 | White | NonMet | No | T1b NX MX |  |  | 1 | 14 |
| 115 | BR112-139-T |  |  |  | NonMet |  |  | N/A | III |  |  |
| 116 | BR112-144-T | M | 57 | White | NonMet | No | T3 NX MX | N/A | II | 1.3 | 18 |
| 117 | BR107-64-1N | F | 42 | White | NonMet | No | T1a NX MX |  |  | 0.6 | 11 |
| 117 | BR107-64-1T |  |  |  | NonMet |  |  | N/A | II |  |  |
| 119 | BR112-171-1T | M | 72 | White | NonMet | No | T1b NX MX | Present | IV | 0.9 | 14 |
| 120 | BR112-143-T (T2) | M | 76 | White | NonMet | No | T1a NX MX | N/A | II | 0.7 | 15 |
| 121 | BR112-172-1T | M | 66 | Black | NonMet | No | T1b NX MX | N/A | II | 2.1 | 44 |
| 124 | BR112-131-T | F | 77 | White | NonMet | No | T1a NX MX | N/A | II | 0.6 | 19 |
| 125 | BR107-55 | M | 53 | Unknown | Met | No | T3 N0 MX | N/A | III | 1.2 |  |
| 127 | BR112-135-N | M | 50 | White | NonMet | No | N/A |  |  | 1 | 11 |
| 127 | BR112-135-T |  |  |  | NonMet |  |  | N/A | N/A |  |  |
| 128 | BR112-129-N | M | 64 | White | NonMet | No | T1a NX MX |  |  | 1.1 | 30 |
| 128 | BR112-129-T |  |  |  | NonMet |  |  | N/A | II |  |  |
| 129 | BR112-132-N | M | 59 | White | Met | No | T1a NX MX |  |  | 0.8 | 28 |
| 129 | BR112-132-T |  |  |  | Met |  |  | N/A | II |  |  |
| 130 | 56-T (1) | M | 51 | Black | Met | No | T3a N0 MX | 50-75% |  | 1.1 | 20 |
| 130 | BR101-56 (4) |  |  |  | Met |  |  | N/A | III |  |  |
| 132 | BR112-173-1T | F | 76 | White | NonMet | No | T1b NX MX | N/A | II/IV | 0.6 | 26 |
| 135 | BR107-15 | F | 44 | White | NonMet | No | T1b NX MX | N/A | II/IV | 0.5 | 8 |
| 136 | BR112-174-1T | F | 72 | N/A | NonMet | No | T1a NX MX | N/A | II/IV | 0.8 | 19 |
| 137 | BR112-099-T | M | 61 | White | NonMet | No | T1a NX MX | N/A | II/IV | 0.8 | 15 |
| 137 | 099-N |  | N/A | N/A | NonMet |  |  |  |  |  |  |
| 138 | BR112-188-1T | N/a | N/A | N/A | Met | No |  | SEEN |  |  |  |
| 139 | 9-T (2) | M | 69 | White | NonMet | No | T3 NX MX | N/A | II | 0.9 | 22 |
| 142 | BR107-10 | N/A | N/A | N/A | NonMet | No | T3b NX MX | N/A | II/IV | 0.9 | 23 |
| 143 | BR107-24 | F | 67 | White | NonMet | No | T1b NX MX | N/A | II | 0.8 | 18 |
| 145 | 54-T (1) | M | 51 | White | Met | No | T2 NX MX | N/A | II | 1.2 | 16 |
| 147 | 45-T (1) | M | N/A | Not Available | Met | No | T3a NX MX | N/A | IV | 0.9 | 21 |
| 148 | BR112-175-1T | N/A | N/A | N/A | NonMet | No | T2 NX MX | N/A | II/IV | 3.5 | 21 |
| 150 | BR107-51 | N/A | N/A | N/A | Met | No | T3a NX M1 | N/A | II-III/IV | 1 | 25 |
| 152 | BR107-47 | M | 51 | Black | Met | No | T3a N1 M1 | SEEN | IV/IV | 0.8 | 9 |
| 153 | BR112-100-T | N/A | N/A | N/A | NonMet | No | T1b NX MX | N/A | II/IV | 0.7 | 14 |
| 153 | 100-N |  |  | N/A | NonMet |  |  |  | N/A |  |  |
| 155 | BR107-26 | F | 73 | Asian | NonMet | No | T1b NX MX | NEGATIVE | IV/IV | 0.9 | 18 |
| 157 | BR107-19-N | F | 49 | White | NonMet | No | T1b NX MX |  |  | 0.7 | 21 |
| 157 | BR107-19-T |  |  |  | NonMet |  |  | N/A | I-II |  |  |
| 158 | BR112-093-T | F | 68 | White | NonMet | No | T1b NX MX | N/A | III/IV, I, II-III | 0.9 | 16 |
| 159 | BR142-191-T | M | 69 | White | NonMet | No | T1a NX MX | N/A | III/IV | 1 | 19 |
| 161 | BR107-13-N | M | 79 | White | NonMet | No | T2 NX MX |  |  | 0.9 | 19 |
| 161 | BR107-13-T |  |  |  | NonMet |  |  | N/A | III/IV |  |  |
| 162 | BR107-41 | M | 56 | White | NonMet | No | T3a NX MX | N/A | II/IV | 1.2 | 15 |
| 163 | BR107-46 | M | 59 | White | Met | Yes | T4 N1 M1 | EXTENSIVE | IV/IV | 1.3 | 15 |
| 164 | BR112-094-T | F | 68 | Black | NonMet | No | T1b N0 MX | N/A | II/IV | 1.22 | 14 |
| 164 | 094-N |  |  | N/A | NonMet |  |  |  |  |  |  |
| 168 | BR107-34 | Not Available | N/A | N/A | NonMet | No | T3a NX MX | N/A | II | 13.4 | 60 |
| 169 | BR112-189-1T | M | 75 | Black | NonMet | No | T1a NX MX | N/A | III/IV | 1 | 19 |
| 170 | BR112-176-1T | F | 63 | White | NonMet | No | T1a NX MX | N/A | II | 0.9 | 11 |
| 171 | BR101-012 (2) | M | 46 | White | NonMet | No | T3b N0 MX |  |  | 0.9 | 13 |
| 171 | 12-T (7) |  | N/A | N/A | NonMet |  |  | SEEN | IV/IV |  |  |
| 172 | BR112-101-T | F | 50 | White | NonMet | No | T1a NX MX | N/A | IV/IV | 0.7 | 9 |
| 173 | BR112-102-T Rebx | M | 45 | Black | NonMet | No | T1a NX MX | N/A | II/IV | 16.49 | 78 |
| 174 | BR112-103-T | N/A | N/A | N/A | NonMet | No | T2 NX MX | N/A |  | 0.6 | 15 |
| 174 | 103-N |  |  |  | NonMet |  |  |  | II |  |  |
| 176 | BR112-104-T | N/A | N/A | N/A | NonMet | No | T1a NX MX | N/A | II | 1.1 | 17 |
| 181 | BR112-105-T | M | 63 | Black | NonMet | No | T1a NX MX | N/A | II/IV | 1 | 9 |
| 182 | BR112-106-T | M | 75 | White | NonMet | No | T1a NX MX | N/A | III/IV | 0.8 | 17 |
| 185 | BR112-107-T | F | 36 | White | NonMet | No | T1a NX MX | N/A | II | 0.8 | 11 |
| 185 | 107-N |  | N/A |  | NonMet |  |  |  |  |  |  |
| 189 | BR112-157-1T | M | 67 | White | NonMet | No | T1a NX MX | N/A | II | 0.8 | 12 |
| 193 | BR112-156-1T | M | 62 | Black | NonMet | No | T3b NX MX | N/A | II/IV | 1.4 |  |
| 194 | BR112-162-T | M | 73 | White | NonMet | No | T1a NX MX | N/A | II | 1.1 | 14 |
| 195 | BR112-179-1T | M | 47 | White | NonMet | No | T1b NX MX | N/A | II/IV | 1.3 | 16 |
| 199 | BR112-108-T | M | 57 | White | NonMet | No | T3a NX MX | PRESENT | IV | 1 | 17 |
| 199 | 108-N |  |  | N/A | NonMet |  |  |  |  |  |  |
| 200 | BR112-095-T | M | 51 | White | Met | No | T1a N0 MX | N/A | I | 0.8 | 10 |
| 200 | 095-N |  |  | N/A | Met |  |  |  |  |  |  |
| 201 | BR112-109-T | F | 67 | White | NonMet | No | T1b NX MX | N/A | II-III | 0.7 | 12 |
| 205 | BR112-096-T | M | 65 | White | NonMet | No | T1a NX MX | N/A | II | 0.9 | 12 |
| 206 | BR112-180-1T | F | 74 | Black | NonMet | No | T1b NX MX | N/A | II | 1.68 | 39 |
| 207 | BR112-134-N | F | 58 | Black | NonMet | No | T1a NX MX |  |  | 0.9 | 16 |
| 207 | BR112-134-T |  |  |  | NonMet |  |  | N/A | II/IV |  |  |
| 208 | BR112-097-T | F | 78 | Black | NonMet | No | T3a N0 MX | NEGATIVE | III/IV | 0.6 | 13 |
| 208 | 097-N |  |  |  |  |  | N/A |  |  |  |  |
| 209 | BR112-110-T | F | 55 | Asian | NonMet | No | T1a NX MX | N/A | II | 0.6 | 10 |
| 209 | 110-N |  |  |  | NonMet |  | N/A |  |  |  |  |
| 212 | BR107-17 | M | 72 | White | NonMet | No | T3 NX MX | N/A | III | 0.8 | 19 |
| 213 | BR112-181-1T | M | 40 | Black | NonMet | No | T1a NX MX | N/A | II | 8.5 | 30 |
| 214 | BR112-182-1T | M | 62 | White | NonMet | No | T3a N0 MX | N/A | III | 1.8 | 26 |
| 215 | BR112-183-1T | M | 74 | White | NonMet | No | T2 NX MX | N/A | II/IV | 0.85 | 12 |
| 218 | 111-N | M | 67 | White | NonMet | No | T1a NX MX | N/A | II | 0.89 | 11 |
| 220 | BR112-112-T | F | 66 | Black | NonMet | No | T3a N0 MX | PRESENT | II | 1.2 | 24 |
| 221 | BR112-113-T | F | 66 | White | NonMet | No |  | N/A | N/A | 0.5 | 13 |
| 221 | 113-N |  |  |  |  |  | N/A |  |  |  |  |
| 222 | BR112-190-1T | M | 36 | White | Met | No | N/A | N/A | N/A |  |  |
| 225 | BR142-192-T | M | 56 | White | NonMet | No | T1a NX MX | N/A | II/IV | 0.6 | 6 |
| 228 | BR112-114-T | M | 71 | White | NonMet | No | T3a NX MX | N/A | III | 1 | 19 |
| 229 | BR107-20 | M | 65 | White | NonMet | No | T2 NX MX | N/A | III/IV | 0.9 | 14 |
| 231 | BR112-115-T | M | 73 | Unknown | NonMet | No | T1a NX MX | N/A | II/IV | 1 | 12 |
| 231 | 115-N |  | N/A |  | NonMet |  |  |  |  |  |  |
| 233 | BR112-185-1T | F | 56 | White | NonMet | No | T3a N0 MX | N/A | III | 1.77 | 39 |
| 234 | BR142-193-T | M | 66 | White | NonMet | No | T1b NX MX | N/A | II/IV | 0.7 | 14 |
| 236 | BR112-184-1T | M | 70 | Black | NonMet | No | T1a NX MX | N/A | II | 0.9 |  |
| 239 | BR142-195-T | F | 60 | Black | NonMet | No | TX NX MX | N/A |  | 0.8 | 12 |
| 242 | BR142-196-T | F | 70 | White | NonMet | No | T1a NX MX | N/A | II | 0.6 | 18 |
| 244 | BR142-197-T | N/A | N/A | N/A | Met | No | T4 N0 M1 | N/A | IV/IV | 0.9 | 12 |
| 248 | BR142-198-T | M | 79 | White | Met | No | T1b N0 MX | N/A | II | 0.87 | 21 |
| 250 | BR142-199-T | M | 59 | White | Met | No | N/A | N/A |  |  |  |
| 251 | BR107-58-N | M | 60 | White | Met | No | T3b N0 MX |  |  | 0.6 | 11 |
| 251 | BR107-58-T |  |  |  | Met |  |  | PRESENT | IV |  |  |
| 252 | BR107-50 (2) | M | Not Available | N/A | Met | No | T3b N0 MX |  |  | 1.29 | 19 |
| 252 | 50(3)-T |  |  |  | Met |  |  | N/A | I/IV |  |  |
| 257 | BR101-44 | M | 67 | White | Met | No | T3 N3 MX | N/A | II/III | 1.1 | 17 |
| 258 | BR107-70 | N/A | Not Available | N/A | NonMet | No | T1b NX MX | N/A | II | 11 | 0.5 |
| 259 | BR107-001 | F | 59 | White | NonMet | No | T1b NX MX | N/A | II | 0.5 | 15 |
