# Supplementary material for: Metabolomic profiling of renal cell carcinoma to identify prognostic markers of metastasis
Source: PLoS One. 2026 Nov 11;21(11):e0361404. doi: 10.1371/journal.pone.0361404 (PMC13754214; doi:10.1371/journal.pone.0361404)
Supplement: Supplementary file 2 [file pone.0361404.s003.docx]

| **PID** | **CTPS ID** | **Site** | **Surgery** | **Comorbidities** | **Treatment** |
| --- | --- | --- | --- | --- | --- |
|  | RT1012 | Edm | Cytoreductive nephrectomy | hypertension, an episode in the past of significant cellulitis in left lower extremity which left with some neuropathic pain, hyperlipidemia, had to have a splenectomy at the same time as nephrectomy, | Sunitinib(50)-Renal-CC; Pazopanib-Renal-Prov; external beam radiation therapy |
|  | RT1054 | Edm | Laparoscopic left radical nephrectomy | Hypertension. Atrial fibrillation. Diabetes mellitus - well controlled on metformin. Diabetic neuropathy involving left foot with reduced sensation distally. | clinical trial looking at individualized dosing of sunitinib |
|  | RT1165 | Edm | right-sided radical nephrectomy | Prolapsed disc requiring discectomy in his lower back, Hypothyroidism. | Sunitinib(50)-Renal-CC |
|  | RT1455 | Edm | radical right nephrectomy | dyslipidemia, takes Crestor, a history of VIN III lesions, underwent excision, hypertension, used to take antihypertensive medications | T12 vertebral body is stable from high dose of palliative radiotherapy |
|  | RT1499 | Edm | left laparoscopic radical nephrectomy | Significant for hypertension | surgical resection of adrenal gland, femur reconstruction with beam radiotherapy, Pazopanib-Renal-Prov for renal metastasis treatment |
|  | RT1509 | Edm | nephrectomy | significant for hypertension, history of epilepsy | metastatectomy, en bloc resection. Pazopanib-Renal-Prov, Sunitinib(50)-Renal-CC, Axitinib-Renal-Prov, Nivolumab(q2wk)-Renal-Prov, Palliative radiation therapy |
|  | RT1584 | Edm | nephrectomy | not available | thoracotomy for metastatic disease, Pazopanib-Renal-Prov, Sunitinib(25)-Renal-CC |
|  | RT1629 | Edm | laparoscopic nephrectomy | Is notable for gout | renal Ca-No treatment except surgery |
|  | RT1727 | Edm | radical nephrectomy | fit individual and otherwise healthy | Sunitinib(50)-Renal-CC, |
|  | RT1828 | Edm | Radical nephrectomy left kidney | Hypertension. Significant trauma from motor vehicle accidents in the past. | Craniotomy for left occipital brain metastases, Palliative whole-brain irradiation, Palliative radiation to right iliac crest and left SI joint, RT whole brain plus 5 fractions to sacrum, Sunitinib(50)-Renal-CC, Axitinib-Renal-Prov, Everolimus-Renal-Prov, Everolimus-Renal-Prov, |
|  | RT832 | Edm | radical nephrectomy | unremarkable | Sunitinib(50)-Renal-CC, Everolimus-Renal-Prov |
|  | RT858 | Edm | laparoscopic right nephrectomy | significant for hypothyroidism and hypertension | Sunitinib(50)-Renal-CC |
|  | RT862 | Edm | laparoscopic radical nephrectomy | unremarkable | Pazopanib-Renal-Prov, Sunitinib(50)-Renal-CC, Everolimus-Renal-Prov |
|  | UT458 | Edm | Left total radical nephrectomy | IgG Kappa multiple myeloma, | Nothing for Renal except surgery, For myeloma, High dose Melphalan, stem cell transplant for myeloma, Velcade, cyclophosphamide, and alternate-day prednisone, Relapse, with mild hypercalcemia, Current therapy of Velcade, cyclophosphamide, and steroids for control of what now has become a virtually nonsecretory-type multiple myeloma |
|  | UT486 | Edm | right nephrectomy- laparoscopic approach | no significant family history pertaining to the renal cell carcinoma | Interferon alpha 2a, Sorafenib, Sunitinib, AZD-2171 study with Rifampin, palliative radiotherapy to the left femur |
|  | UT517 | Edm | right renal nephrectomy | Hypertensive encephalopathy, Hypertension | elected to have treatment elsewhere |
|  | UT576 | Edm | partial nephrectomy | Ankylosing spondylitis, Ulcerative colitis treated with a total colectomy, Insomnia, Benign prostatic hypertrophy. | Benign prostatic hypertrophy., Axitinib-Renal-Prov, radiation treatment |
|  | UT583 | Edm | left radical nephrectomy | bilateral lower limb edema, skin breakdown and weeping, diffuse erythematous maculopapular rash | Radiation therapy to L2, SUTENT two cycles ,Posterior left transpedicular resection of renal cell tumour at L2, T12 - L3 instrumented fusion |
|  | UT609 | Edm | left nephrectomy | Crohn’s disease, total colectomy, a small bowel resection | Sunitinib(50)-Renal-CC for primary renal Ca |
|  | UT626 | Edm | surgically removed | Negative, an appendectomy long ago | renal Ca-No treatment except surgery |
|  | UT646 | Edm | recommended a radical nephrectomy | hyperlipidemia, multiple sclerosis, hypertension and hypothyroidism | not available |
|  | UT733 | Edm | nephrectomy | Essentially unremarkable. Hypertension | thoracotomy for resection, Sunitinib, Pazopanib, nivolumab, Cabozantinib |
|  | UT742 | Edm | not available | not available | not available |
|  | UT761 | Edm | nephrectomy | degenerative arthritis, a sensitive stomach reacts with nausea and vomiting to a variety of agents including anesthetics and analgesics | renal Ca-No treatment except surgery |
|  | UT769 | Edm | left radical nephrectomy | history of some fractures and trauma in the past | clinical trial, NCIC-REC.2 but discontinued treatment after 1 month due to toxicity |
|  | UT837 | Edm | Partial nephrectomy | High blood pressure | renal Ca-No treatment except surgery |
|  | UT865 | Edm | not available | not available | not available |
|  | UT871 | Edm | left kidney, treated with a radical nephrectomy | hypertension and hypothyroidism | beam radiotherapy |
|  | F10012 | Cgy | Radical nephrectomy | colon adenocarcinoma/resection the same day as kidney procedure, osteoporosis and cataracts, previous bilateral breast cysts, only medication on admission was alendronate, | renal Ca-No treatment except surgery |
|  | R105 | Cgy | Right Kidney, Nephrectomy | very active without any limitation. | renal Ca-No treatment except surgery |
|  | R236 | Cgy | Right Kidney, Partial Nephrectomy | Hypertension | renal Ca-No treatment except surgery |
|  | R238 | Cgy | Left Kidney, Radical Nephrectomy | been quite well all life | renal Ca-No treatment except surgery, no treatment for metastasis |
|  | R279 | Cgy | Right Kidney, Partial Nephrectomy | slight high blood pressure on diuretic, hemorrhoidectomy | Sunitinib-Int(50)-Renal-Prov, Nivolumab(q2wk)-Renal-Prov, Cabozantinib-Renal-Prov, radiation, |
|  | R420 | Cgy | Left Kidney, Radical Nephrectomy | patient is very healthy | multiple surgery for metastases |
|  | R440 | Cgy | Left Kidney, Radical Nephrectomy | no mention except melanoma which was detected at later date | renal Ca-No treatment except surgery |
|  | R487 | Cgy | Right Kidney, Radical Nephrectomy | bronchitis approximately 2 years ago | renal Ca-No treatment except surgery |
|  | R525 | Cgy | Left Kidney, Partial Nephrectomy | prostatectomy, kidney stone basket extraction, pneumothorax but has had no recurrence, tonsils removed | renal Ca-No treatment except surgery, no treatment for metastasis |
|  | R56 | Cgy | Right Kidney, Radical Nephrectomy | Malignant neoplasm of other and unspecified testis | underwent cryotherapy |
|  | R599 | Cgy | Right Renal Tumor, Partial Nephrectomy | heart having a silent MI, known hypertensive, concentric LVH with also moderate aortic stenosis, a history of chronic renal failure, hypothyroidism, long history of chronic depression, hematological issue with MIG Kappa monoclonal gammopathy | renal Ca-No treatment except surgery |
|  | R85 | Cgy | (Left) Radical Nephrectomy | not available | Everolimus-Renal-Prov, Axitinib-Renal-Prov, radiotherapy |
|  | R87 | Cgy | Kidney, Left, Radical Nephrectomy | diet controlled diabetes, occasional migraine headaches, hypertension and hypercholesterolemia | renal Ca-No treatment except surgery |
|  | **GUTB** |  |  |  |  |
|  | 284-A | Tx | Left radical nephrectomy | CKD stage IV, hypothyroidism, hypertension, hypercholesterolemia, diabetes type II | N/A |
|  | 294-A | Tx | Right partial nephrectomy | Hypercholesterolemia, diabetes type II | N/A |
|  | 296-D | Tx | Right radical nephrectomy | CAD,Hypercholesterolemia, Osteoarthritis, Hypertension, | N/A |
|  | 297-A | Tx | Left partial nephrectomy | CAD, Prostate cancer, hypertension, osteroarthritis | N/A |
|  | 298-A | Tx | Right partial nephrectomy | Hypertension, Hepatitis C | N/A |
|  | 300-A.2 | Tx | Left partial nephrectomy | Diabetes Type II, hypertension, hyperlipidemia, breast cancer, CAD, CKD stage 3 | N/A |
|  | 301-A.2 | Tx | Right radical nephrectomy | Diabetes Type II, Schizophrenia | N/A |
|  | 302-C.2 | Tx | Right Partial Nephrectomy | hypertension, diabetes type II, hyperlipidemia | N/A |
|  | 304-E.A.2 | Tx | Right Partial Nephrectomy, right-posterior renal mass | osteoarthritis, BCC of skin, hyperlipidemia, hypertension | N/A |
|  | 305-A.2 | Tx | Left partial nephrectomy | Hyperlipidemia, osterarthritis, hypertension, Hep C, COPD | N/A |
|  | 308-C.2 | Tx | Right radical nephrectomy | diabetes type II, hyperlipidemia, hypertension,CAD | N/A |
|  | 310-C.E.2 | Tx | Rigth radical nephrectomy, mass on superior pole, extends to lower pole, 3/4's of kidney | COPD, hyperlipidemia | N/A |
|  | 319-C | Tx | Left partial nephrectomy | hypertension, hyperlipidemia, | N/A |
|  | 289-A | Tx | Right partial nephrectomy | hypertension, hypercholesterolemia, CKD stage 3, diabetes type II | N/A |
|  | 292-F | Tx |  | hypertension, | N/A |
|  | 295-F | Tx | Right radical nephrectomy | Hypercholesterolemia, osteroarthiritis, Diabetes type II | N/A |
|  | 307-U.2 | Tx | Left open nephrectomy, 9 cm renal mass | COPD, dibetes type II, hypertension, osterarthritis | N/A |
| **PID** | **EVMS Bio** |  |  |  |  |
| 1 | BR101-48 | Va | Radical Nephrectomy | HTN , CAD , Splenectomy , Hypercholesterolemia , Diabetes Mellitus , Dyslipidemia | failed sunitinib, then was on torisel |
| 3 | BR101-42 | Va | Radical Nephrectomy | Hysterectomy , TMJ Syndrome | radiation, neoadjuvant and adjuvant chemo with sunitinib |
| 4 | 29-T (5) | Va | Radical Nephrectomy | HTN , Brain Mass | clinical trial using sunitinib, XRT |
| 9 | BR101-27-N | Va | Radical Nephrectomy | Obesity , HTN , Hypercholesterolemia | sunitinib |
| 9 | BR101-27-T | Va |  |  |  |
| 10 | BR101-49 | Va | Radical Nephrectomy | Lung Cancer, Knee Surgery | 6 lines of therapy, sunitinib, everolimus, axitinib, nivolumab |
| 11 | BR107-69-N | Va | Laparoscopic Radical Nephrectomy | HTN , Hypercholesterolemia | Resection Only |
| 11 | BR107-69-T | Va |  |  |  |
| 12 | BR112-119-T | Va | Laparoscopic Radical Nephrectomy | HTN , Hypercholesterolemia , Mental/Nervous Disorder , Hernia Repair , Cholecystectomy | Resection Only |
| 12 | 119-N | Va |  |  |  |
| 13 | 3-T (6) | Va | Laparoscopic Radical Nephrectomy | Hypercholesterolemia , HTN , Thyroid Disease , Obesity , Breast Lumpectomy , Cholecystectomy | On Lenvatinib and Pembrolizumab |
| 18 | BR101-2 | Va | Laparoscopic Radical Nephrectomy | Hernia Repair , HTN , Hypercholesterolemia | Resection Only |
| 19 | BR101-16-N | Va | Laparoscopic Radical Nephrectomy | None listed | Resection Only |
| 19 | BR101-16-T | Va |  |  |  |
| 20 | 120-N | Va | Partial Nephrectomy | HTN , Stroke , Coronary Artery Disease (CAD) , Hypercholesterolemia | Resection Only |
| 21 | BR112-121-T | Va | Partial Nephrectomy | HTN , Diabetes Mellitus , Occular Melanoma , Kidney Stones | Not Available |
| 21 | 121-N | Va |  |  |  |
| 22 | 61-N (5) | Va | Laparoscopic Radical Nephrectomy | HTN , Borderline Hyperlipidemiai , mild obesity | Resection Only |
| 22 | 61-T (6) | Va |  |  |  |
| 23 | BR101-28 | Va | Radical Nephrectomy | HTN , Hypothyroidism , Hemodialysis , Cholecystectomy , Appendectomy , Pancreatomy | Resection Only |
| 24 | BR101-4 | Va | Laparoscopic Radical Nephrectomy | Rectal cancer | adrenalectomy |
| 29 | BR107-65 | Va | Laparoscopic Radical Nephrectomy | Bipolar Disorder , Tonsillectomy , Septoplasty | Resection Only |
| 30 | BR107-43 | Va | Radical Nephrectomy | Colon cancer , Hyperlipidemia , Partial Colectomy , Appendectomy | sunitinib -> affinator -> nexovar -> torisel |
| 31 | BR112-136-N | Va | Radical Nephrectomy | Hysterectomy | Resection Only |
| 31 | BR112-136-T | Va |  |  |  |
| 32 | 116-N | Va | Laparoscopic Radical Nephrectomy | Hypercholesterolemia , HTN , Hernia Repair , Appendectomy , Lumbar Laminectomy , Hyperlipidemia , Pacemaker | Resection Only |
| 33 | BR107-87-N | Va | Partial Nephrectomy | Breast Bx-benign , Hypercholesterolemia | Resection Only |
| 33 | BR107-87-T | Va |  |  |  |
| 34 | BR107-63-1T | Va | Laparoscopic Partial Nephrectomy | HTN , C-section | Resection Only |
| 35 | BR107-67 | Va | Radical Nephrectomy | Hyperthyroidism | Resection Only |
| 36 | BR107-59-N | Va | Radical Nephrectomy | None listed | Resection Only |
| 36 | BR107-59-T | Va |  |  |  |
| 37 | BR112-148-T | Va | Radical Nephrectomy | HTN , Hypercholesterolemia , Hysterectomy | Resection Only |
| 38 | BR112-151-T | Va | Partial Nephrectomy | HTN , Hypercholesterolemia , CAD , Sleep Apnea , Hernia Repair | clinical trial using sunitinib -> chemotherapy |
| 39 | BR107-86 | Va | Radical Nephrectomy | Mixed Hyperlipidemia , Skin cancer , Lung Nodule , HTN , Diabetes Mellitus | on initial Votrient and subsequent Nuvolumab, more recently on everolimus |
| 40 | BR107-89 | Va | Partial Nephrectomy | HTN , Hyperlipidemia , Hypercholesterolemia , Diabetes Mellitus | Resection Only |
| 41 | BR107-52-N | Va | Radical Nephrectomy | HTN , Prostate Cancer | sunitinib, radiation/chemotherapy |
| 41 | BR107-52-T | Va |  |  |  |
| 42 | BR112-177-1T | Va | Laparoscopic Radical Nephrectomy | Diabetes Mellitus , HTN , Cardiac Disease | Unknown |
| 43 | BR112-057-1T | Va | Radical Nephrectomy | Tubal Ligation , HTN | Resection Only |
| 46 | BR107-80-N | Va | Partial Nephrectomy | HTN , Hypercholesterolemia , Coronary Artery Disease (CAD) , Appendectomy | Resection Only |
| 46 | BR107-80-T | Va |  |  |  |
| 48 | BR112-178-1T | Va | Laparoscopic Radical Nephrectomy | Hypercholesterolemia , HTN , Diabetes Mellitus , Hysterectomy | sunitinib -> torisel -> sorafineb |
| 49 | BR112-186-1T | Va | Radical Nephrectomy | None listed | Resection Only |
| 51 | BR112-164-1T | Va | Laparoscopic Radical Nephrectomy | HTN , Heart Disease , Coronary Artery Disease (CAD) | Resection Only |
| 52 | BR112-145-T | Va | Laparoscopic Radical Nephrectomy | Hernia | Resection Only , radiation therapy, started on cabozantinib |
| 53 | BR112-149-T | Va | Radical Nephrectomy | Diabetes Mellitus , Thyroid Cancer , Cholecystectomy , Hiatus Hernia Syndrome , Obesity , Thyroidectomy , C-section | Resection Only |
| 55 | BR107-85-N | Va | Laparoscopic Radical Nephrectomy | None listed | radiotherapy |
| 55 | BR107-85-T | Va |  |  |  |
| 56 | BR107-91-N | Va | Radical Nephrectomy | Breast cancer , HTN , Breast Lumpectomy , Stroke | Resection Only |
| 56 | BR107-91-T | Va |  |  |  |
| 57 | BR107-81-N | Va | Partial Nephrectomy | HTN , Hypercholesterolemia | Resection Only |
| 57 | BR107-81-1T | Va |  |  |  |
| 58 | BR112-150-T | Va | Partial Nephrectomy | Diabetes Mellitus Type II | Resection Only |
| 59 | BR112-187-1T | Va | Partial Nephrectomy | HTN , Hypercholesterolemia , Diabetes Mellitus | radiation therapy, pembrolizumab + axitinib -> cabozantinib -> pembrolizumab |
| 60 | BR112-147-T | Va | Radical Nephrectomy | HTN | sunitinib |
| 62 | BR112-124-T | Va | Radical Nephrectomy | HTN , Hypercholesterolemia , Obesity , C-section , Hysterectomy-Partial , Appendectomy , Hernia Repair , Oophorectomy | Resection Only |
| 66 | BR112-125-T | Va | Radical Nephrectomy | HTN , AAA , Colon cancer , Erectile Dysfunction | Unknown |
| 67 | BR107-78-N | Va | Partial Nephrectomy | HTN , Hypercholesterolemia , Diabetes Mellitus , Pacemaker | Resection Only |
| 67 | BR107-78-T | Va |  |  |  |
| 68 | BR107-76 | Va | Partial Nephrectomy | Tonsillectomy , HTN , Diabetes Mellitus Type II , Umbilical Hernia repair , Hyperlipidemia | Resection Only |
| 70 | BR112-152-T | Va | Partial Nephrectomy | Hyperlipidemia , Cholecystectomy | Resection Only |
| 71 | BR112-127-T(T2) | Va | Radical Nephrectomy | fx vertebra , Hysterectomy , Esophageal Reflux | sunitinib, opdivo, toricel,chemotherapy |
| 72 | BR112-128-T | Va | Laparoscopic Radical Nephrectomy | HTN , Hypercholesterolemia , cardiac dysrhythmia , Heart Surgery , Appendectomy , Obesity | Resection Only |
| 72 | 128-N | Va |  |  |  |
| 73 | BR112-159-T | Va | Partial Nephrectomy | Asthma , Hypercholesterolemia , HTN , Hysterectomy | Resection Only |
| 74 | BR107-83 | Va | Radical Nephrectomy | HTN , Hyperlipidemia | suboccipital craniotomy/cyberknife XRT |
| 75 | BR142-075-T | Va | Laparoscopic Radical Nephrectomy | HTN , Erectile Dysfunction | Resection Only |
| 76 | BR107-73 | Va | Laparoscopic Radical Nephrectomy | gastric ulcer , Hysterectomy , C-section | Resection Only |
| 80 | BR112-165-1T | Va | Radical Nephrectomy | Hernia Repair , Esophageal Reflux , Tubal Ligation | Resection Only |
| 81 | 7-N (5) | Va | Radical Nephrectomy | HTN , Hypercholesterolemia , Orthopedic Surgery | Resection Only |
| 81 | 7-T (6) | Va |  |  |  |
| 84 | BR112-166-1T | Va | Radical Nephrectomy | HTN , Morbid Obesity , Renal Failure , Cocaine Abuse , Marijuana Abuse | sunitinib -> everolimus -> axitinib -> pazopanib -> opdivo -> everolimus |
| 85 | BR112-167-1T | Va | Laparoscopic Radical Nephrectomy | HTN , Hypercholesterolemia | Resection Only |
| 86 | BR107-79-N | Va | Laparoscopic Radical Nephrectomy | Hypothyroidism , Hypercholesterolemia , GERD , C-section | Resection Only |
| 86 | BR107-79-T | Va |  |  |  |
| 87 | BR112-098-T (T2) | Va | Partial Nephrectomy | diabetes mellitus , HTN , Hypercholesterolemia , Coronary Artery Disease (CAD) | Resection Only |
| 89 | BR107-30-N | Va | Laparoscopic Radical Nephrectomy | Thyroid Disease , Hyperlipidemia , HTN , Sciatica | Resection Only |
| 89 | BR107-30-T | Va |  |  |  |
| 91 | BR112-161-T | Va | Partial Nephrectomy | HTN , diabetes mellitus , Tubal Ligation , Oophorectomy | Resection Only |
| 92 | BR107-053 (T3) | Va | Laparoscopic Radical Nephrectomy | Diabetes Mellitus Type II , HTN , Hypothyroidism | Unknown |
| 92 | BR107-53 | Va |  |  |  |
| 94 | BR107-005 | Va | Radical Nephrectomy | HTN , Coronary Artery Disease (CAD) | Resection Only |
| 96 | BR112-126-1T | Va | Radical Nephrectomy | Hypercholesterolemia , HTN , Hyperlipidemia , Hypervolemia | Resection Only |
| 97 | BR112-169-2T | Va | Laparoscopic Radical Nephrectomy | Thyroid Disease , Hysterectomy | sunitinib -> everolimus, chemotherapy |
| 98 | BR107-71 | Va | Radical Nephrectomy | Hypothyroidism , Thyroid Disease , Cholecystectomy | Resection Only |
| 99 | 14-N (2) | Va | Laparoscopic Radical Nephrectomy | diabetes mellitus , HTN , Hyperlipidemia , Hysterectomy , Appendectomy | Resection Only |
| 99 | 14-T (5) | Va |  |  |  |
| 100 | BR112-137-T | Va | Laparoscopic Radical Nephrectomy | HTN , Thyroid Cancer , Hypercholesterolemia , Hysterectomy , Diabetes Mellitus Type II | Resection Only |
| 103 | BR107-90 | Va | Radical Nephrectomy | HTN , Erectile Dysfunction , Prostate Cancer , Coronary Artery Disease (CAD) | Resection Only |
| 109 | BR107-008-N | Va | Radical Nephrectomy | HTN , Hypothyroid , Vasectomy | Resection Only |
| 109 | BR107-008-T | Va |  |  |  |
| 110 | BR112-141-T | Va | Radical Nephrectomy | HTN , Hypercholesterolemia , Hernia Repair | Resection Only |
| 111 | BR112-138-N | Va | Laparoscopic Radical Nephrectomy | HTN | Resection Only |
| 111 | BR112-138-T | Va |  |  |  |
| 112 | BR112-170-1T | Va | Partial Nephrectomy | HTN , diabetes mellitus , Hypercholesterolemia , Coronary Artery Disease (CAD) | none -> patient passed away shortly after diagnosis of multiple met lesions and was too weak for treatment |
| 114 | BR107-21 | Va | Radical Nephrectomy | HTN , Hernia Repair | Prior Treatments: Pazopanib, single agent nivolumab, cabozantinib, everolimus, Ipilimumab + Nivolumab |
| 115 | BR112-139-N | Va | Partial Nephrectomy | Hernia Repair | Resection Only |
| 115 | BR112-139-T | Va |  |  |  |
| 116 | BR112-144-T | Va | Radical Nephrectomy | HTN , Colon Cancer | radiation therapy, chemotherapy with pembrolizumab and axitinib |
| 117 | BR107-64-1N | Va | Partial Nephrectomy | Depression | Resection Only |
| 117 | BR107-64-1T | Va |  |  |  |
| 119 | BR112-171-1T | Va | Partial Nephrectomy | HTN , Obesity , Mastectomy | Hospice |
| 120 | BR112-143-T (T2) | Va | DV Assisted Laparoscopic Partial Nephrectomy | None listed | Resection Only |
| 121 | BR112-172-1T | Va | DV Assisted Partial Nephrectomy | HTN , Hypercholesterolemia , Cardiac Bypass x2 | Resection Only |
| 124 | BR112-131-T | Va | Partial Nephrectomy | HTN , Hypercholesterolemia , Parathyroid Adenoma , Lipoma , Scoliosis | Resection Only |
| 125 | BR107-55 | Va | Radical Nephrectomy | diabetes mellitus , HTN , Hypercholesterolemia | Resection Only |
| 127 | BR112-135-N | Va | Radical Nephrectomy | None listed | Resection Only |
| 127 | BR112-135-T | Va |  |  |  |
| 128 | BR112-129-N | Va | Laparoscopic Radical Nephrectomy | HTN , diabetes mellitus , Hernia Repair | Resection Only |
| 128 | BR112-129-T | Va |  |  |  |
| 129 | BR112-132-N | Va | Partial Nephrectomy | None listed | Resection Only |
| 129 | BR112-132-T | Va |  |  |  |
| 130 | 56-T (1) | Va | Radical Nephrectomy | HTN, Alcohol Abuse | sunitinib, everolimus, chemotherapy |
| 130 | BR101-56 (4) | Va |  |  |  |
| 132 | BR112-173-1T | Va | Laparoscopic Radical Nephrectomy | HTN , Cholecystectomy , Hypercholesterolemia , Diabetes Mellitus Type II | Hospice |
| 135 | BR107-15 | Va | Laparoscopic Radical Nephrectomy | HTN , Hysterectomy | Resection Only |
| 136 | BR112-174-1T | Va | Radical Nephrectomy | None listed | Resection Only |
| 137 | BR112-099-T | Va | Partial Nephrectomy | brain tumor , HTN , Hyperlipidemia | Resection Only |
| 137 | 099-N | Va |  |  |  |
| 138 | BR112-188-1T | Va |  | HTN , Hernia Repair | Resection Only |
| 139 | 9-T (2) | Va | Radical Nephrectomy | HTN | chemotherapy + pembrolizumab |
| 142 | BR107-10 | Va | Laparoscopic Radical Nephrectomy | diabetes mellitus , heart attack , HTN , Hypercholesterolemia , Cardiac Stent | Resection Only |
| 143 | BR107-24 | Va | Laparoscopic Radical Nephrectomy | HTN , Hysterectomy | Resection Only |
| 145 | 54-T (1) | Va | Cytoreductive Radical Nephrectomy | Hepatitis A , Thyroid Disease , Hepatitis C , Appendectomy | sunitinib -> chemotherapy |
| 147 | 45-T (1) | Va | Cytoreductive Radical Nephrectomy | HTN , Hypercholesterolemia , Coronary Artery Disease (CAD) , Dyslipidemia | Resection Only |
| 148 | BR112-175-1T | Va | Laparoscopic Radical Nephrectomy | HTN , Thyroidectomy , Hyperlipidemia | Resection Only |
| 150 | BR107-51 | Va | Cytoreductive Laparoscopic Radical Nephrectomy | HTN , diabetes mellitus , thyroid dysfunction , Ovarian Cancer , Hysterectomy , Thyroidectomy | Resection Only |
| 152 | BR107-47 | Va | Cytoreductive Radical Nephrectomy | HTN , Hernia Repair | high-dose interleukin-2 |
| 153 | BR112-100-T | Va | Partial Nephrectomy | Breast cancer , HTN , Hypothyroidism , Obesity , Hysterectomy Partial | Resection Only |
| 153 | 100-N | Va |  |  |  |
| 155 | BR107-26 | Va | Laparoscopic Radical Nephrectomy | HTN , diabetes mellitus , Cholecystectomy | Resection Only |
| 157 | BR107-19-N | Va | Laparoscopic Radical Nephrectomy | HTN , High Cholesterol , Hysterectomy | Resection Only |
| 157 | BR107-19-T | Va |  |  |  |
| 158 | BR112-093-T | Va | Laparoscopic Radical Nephrectomy | None listed | Resection Only |
| 159 | BR142-191-T | Va | Partial Nephrectomy | HTN , Hyperlipidemia | Resection Only |
| 161 | BR107-13-N | Va | Radical Nephrectomy | Diabetes Mellitus Type II , HTN , Dyslipidemia , Non-Hodgkins Lymphoma | Axitinib + pembrolizumab, chemotherapy |
| 161 | BR107-13-T | Va |  |  |  |
| 162 | BR107-41 | Va | Radical Nephrectomy | HTN , Hypercholesterolemia , Inguinal Hernia Repair | pembrolizumab + lenvatinib -> axitinib |
| 163 | BR107-46 | Va | Radical Nephrectomy | None listed | radiotherapy |
| 164 | BR112-094-T | Va | Radical Nephrectomy | HTN , Hypercholesterolemia , Hysterectomy | Resection Only |
| 164 | 094-N | Va |  |  |  |
| 168 | BR107-34 | Va | Radical Nephrectomy | Diabetes Mellitus , HTN , Hypercholesterolemia , Hypothyroidism , Coronary Artery Disease (CAD) | Resection Only |
| 169 | BR112-189-1T | Va | DV Assisted Laparoscopic Partial Nephrectomy | None listed | Resection Only |
| 170 | BR112-176-1T | Va | Partial Nephrectomy | HTN | Resection Only |
| 171 | BR101-012 (2) | Va | Radical Nephrectomy | None listed | Resection Only |
| 171 | 12-T (7) | Va |  |  |  |
| 172 | BR112-101-T | Va | Laparoscopic Radical Nephrectomy | Breast cancer, BCC | Resection Only |
| 173 | BR112-102-T Rebx | Va | Laparoscopic Radical Nephrectomy | HTN , ESRD , Hyperparathyroidism | Resection Only |
| 174 | BR112-103-T | Va | Laparoscopic Radical Nephrectomy | HTN , Diabetes , Throat cancer | Resection Only |
| 174 | 103-N | Va |  |  |  |
| 176 | BR112-104-T | Va | Laparoscopic Radical Nephrectomy | HTN , Coronary Artery Disease (CAD) | Resection Only |
| 181 | BR112-105-T | Va | Partial Nephrectomy | None listed | Resection Only |
| 182 | BR112-106-T | Va | Partial Nephrectomy | HTN , Cardiomyopathy , Thyroid Disease , Cholecystectomy | Resection Only |
| 185 | BR112-107-T | Va | DV Assisted Laparoscopic Partial Nephrectomy | None listed | Resection Only |
| 185 | 107-N | Va |  |  |  |
| 189 | BR112-157-1T | Va | Partial Nephrectomy | Diabetes Mellitus Type II , Cholecystectomy | Resection Only |
| 193 | BR112-156-1T | Va | Radical Nephrectomy | None listed | Resection Only |
| 194 | BR112-162-T | Va | Partial Nephrectomy | Cholecystectomy | axitinib + pembrolizumab |
| 195 | BR112-179-1T | Va | Laparoscopic Radical Nephrectomy | None listed | Resection Only |
| 199 | BR112-108-T | Va | Radical Nephrectomy | None listed | Resection Only |
| 199 | 108-N | Va |  |  |  |
| 200 | BR112-095-T | Va | Radical Nephrectomy | HTN , Brain Tumor | XRT -> nivolumab/ipilimumab -> cabozantinib -> radiation therapy -> bevacizumab -> radiation therapy -> pembrolizumab + axitinib |
| 200 | 095-N | Va |  |  |  |
| 201 | BR112-109-T | Va | Laparoscopic Radical Nephrectomy | Diabetes Mellitus Type II , HTN , Hypercholesterolemia , Coronary Artery Disease (CAD) , Hernia Repair , Hysterectomy | Unknown |
| 205 | BR112-096-T | Va | Partial Nephrectomy | Hyperlipidemia , Hypercholesterolemia , Cholecystectomy | Resection Only |
| 206 | BR112-180-1T | Va | Partial Nephrectomy | HTN | Resection Only |
| 207 | BR112-134-N | Va | Partial Nephrectomy | Sickle Cell Trait , HTN , PUD , Bladder Cancer , C-section , Cholecystectomy | Resection Only |
| 207 | BR112-134-T | Va |  |  |  |
| 208 | BR112-097-T | Va | Laparoscopic Radical Nephrectomy | HTN , Coronary Artery Disease (CAD) , Diabetes Mellitus Type II | Resection Only |
| 208 | 097-N | Va |  |  |  |
| 209 | BR112-110-T | Va | Partial Nephrectomy | None listed | Resection Only |
| 209 | 110-N | Va |  |  |  |
| 212 | BR107-17 | Va | Laparoscopic Radical Nephrectomy | None listed | Resection Only |
| 213 | BR112-181-1T | Va | Radical Nephrectomy | HTN , diabetes mellitus , Hyperlipidemia | Resection Only |
| 214 | BR112-182-1T | Va | Radical Nephrectomy | HTN , Diabetes Mellitus Type II , Hypercholesterolemia | Resection Only |
| 215 | BR112-183-1T | Va | Laparoscopic Radical Nephrectomy | HTN , Hyperlipidemia , Diabetes Mellitus Type II | Resection Only |
| 218 | 111-N | Va | Laparoscopic Radical Nephrectomy | None listed | Resection Only |
| 220 | BR112-112-T | Va | Radical Nephrectomy | HTN | XRT |
| 221 | BR112-113-T | Va | Partial Nephrectomy | Breast cancer , Obesity , HTN , Hyperthyroidism | Resection Only |
| 221 | 113-N | Va |  |  |  |
| 222 | BR112-190-1T | Va |  | None listed | pazopanib -> adrenalectomy -> cryoablation -> microwave ablation -> nivolumab + ipilimumab -> cabozantinib -> pembrolizumab + lenvatinib -> chemoembolization |
| 225 | BR142-192-T | Va | Partial Nephrectomy | HTN , Thyroid Cancer | Resection Only |
| 228 | BR112-114-T | Va | Partial Nephrectomy | None listed | Resection Only |
| 229 | BR107-20 | Va | Laparoscopic Radical Nephrectomy | Hypercholesterolemia , HTN , Hyperlipidemia | Resection Only |
| 231 | BR112-115-T | Va | Partial Nephrectomy | HTN | Resection Only |
| 231 | 115-N | Va |  |  |  |
| 233 | BR112-185-1T | Va | Partial Nephrectomy | Hypercholesterolemia , Heart Disease , Breast cancer , CHF , Cholecystectomy | Resection Only |
| 234 | BR142-193-T | Va | Partial Nephrectomy | HTN , Hypercholesterolemia , Skin cancer | Resection Only |
| 236 | BR112-184-1T | Va | Partial Nephrectomy | Prostate Cancer , HTN | Resection Only |
| 239 | BR142-195-T | Va | Partial Nephrectomy | Morbid Obesity , Cardiac Catheter , Cholecystectomy , Hysterectomy , Hernia Repair , Diabetes Mellitus Type II , HTN , high cholesterol | Resection Only |
| 242 | BR142-196-T | Va | Laparoscopic Radical Nephrectomy | HTN , Cholecystectomy | Resection Only |
| 244 | BR142-197-T | Va | Radical Nephrectomy | HTN , Hyperlipidemia | Resection Only |
| 248 | BR142-198-T | Va | Cytoreductive Laparoscopic Radical Nephrectomy | None listed | sunitib -> pazopanib -> chemotherapy |
| 250 | BR142-199-T | Va | Radical Nephrectomy | HTN , Hypercholesterolemia | radiation therapy -> axitinib + pembrolizumab |
| 251 | BR107-58-N | Va | Radical Nephrectomy | HTN, CHF | pazopanib -> opdivo -> chemotherapy |
| 251 | BR107-58-T | Va |  |  |  |
| 252 | BR107-50 (2) | Va | Radical Nephrectomy | HTN , Hypercholesterolemia , Basal Cell Cancer , Coronary Artery Disease (CAD) | Resection Only |
| 252 | 50(3)-T | Va |  |  |  |
| 257 | BR101-44 | Va | Laparoscopic Radical Nephrectomy | HTN , Diabetes Mellitus , Hyperlipidemia , CAD , Hypercholesterolemia , Appendectomy | sorafenib, radiation therapy |
| 258 | BR107-70 | Va | Radical Nephrectomy | HTN , Hyperlipidemia | Resection Only |
| 259 | BR107-001 | Va | Laparoscopic Radical Nephrectomy | Hypercholesterolemia , HTN , Thyroid Disease , Obesity , Breast Lumpectomy , Cholecystectomy | pembrolizumab + lenvatinib |
