# Supplementary material for: Metabolomic profiling of renal cell carcinoma to identify prognostic markers of metastasis
Source: PLoS One. 2026 Nov 11;21(11):e0361404. doi: 10.1371/journal.pone.0361404 (PMC13754214; doi:10.1371/journal.pone.0361404)
Supplement: Supplementary file 3 [file pone.0361404.s004.docx]

Response to Reviewers

Response to Reviewer Comments:

Reviewer #1
-ccRCC is essentially a metabolic disease characterized by a reprogramming of energetic metabolism (PMID: 40082108; PMID: 36960789; PMID: 30983433, PMID: 36430837,PMID: 36310399). In particular the metabolic flux through glycolysis is partitioned (PMID: 30538212; PMID: 29371925, PMID: 28933387, PMID: 25945836), and mitochondrial bioenergetics and OxPhox are impaired, as well as lipid metabolism (PMID: 29371925, PMID: 36430448). In addition, metabolic reprogramming plays an important role in chemoresistance and/or in determining the sensitivity of neoplastic cells to drugs (PMID:41533164).

These findings should be referenced and better discussed.

**Response**: The references have been inserted and briefly discussed in the first para of the Discussion.

Reviewer #2: The manuscript investigates metabolomic profiles of primary ccRCC tumors to identify markers associated with metastatic progression using LC-MS-based chemical isotope labeling and machine learning approaches. The study addresses a clinically relevant question however several aspects require clarification and more cautious interpretation.

1. The Introduction currently lacks a clearly articulated statement of novelty and a concise review of the most relevant LC-MS-based metabolomic studies in RCC (e.g., DOI: 10.1038/s41598-019-52059-y, 10.1186/s12894-025-01767-x, 10.1016/j.eururo.2025.09.4148, 10.1007/s11306-025-02294-4,10.3390/ijms232214360).

**Response**: The references have been added and briefly discussed in the Intro.

2. Key clinical information is missing, including tumor stage and grade, tumor size, presence of tumor necrosis, type of surgical procedure, postoperative treatment, concomitant medications, renal function
and relevant comorbidities.
**Response:** All available clinical data elements are added.

3. The terminology used to describe the study groups is confusing. Please clarify that "controls" refer to non-metastatic ccRCC patients rather than healthy individuals
**Response**: “controls” has been changed to non-metastatic in the text of the paper.

and clearly distinguish patient groups (metastatic vs. non-metastatic tumors) from the adjacent normal tissue samples included in the analysis.
**Response*:*** The text in Materials and Methods has been edited to clarify the patient groups. 3 subject tumors from the Canadian group originally classified as non-metastasis were ultimately staged as M1.

4. Why was extraction performed for 24 h at room temperature rather than using conventional cold extraction protocols?
**Response**: Extraction time and temperature are important considerations. One of our original goals was to establish a work flow for metabolite analysis that would allow histologic examination of the exact same tissue from which we extracted and analyzed metabolites. Rather than impose a requirement for use of cold methanol, which would be somewhat unfamiliar to histology labs, we elected to use room temperature methanol. Our hypothesis is that this provides a more consistent work flow.

Our previous work suggested that room temperature aqueous alcohol does effectively extract metabolites.

We compared standard tissue grinding to simple immersion in aqueous methanol. This showed near complete extraction of metabolites from tissues after 24 hours at room temperature. (Brown MV, McDunn JE, Gunst PR, Smith EM, Milburn MV, Troyer DA, Lawton KA. Cancer detection and biopsy classification using concurrent histopathological and metabolomics analysis of core biopsies. Genome Medicine 2012, 4:33 doi:10.1186/gm33).

Other studies have shown that extraction at -20 ^o^C, room temperature, and 60 ^o^C have been found to be comparable. (Beltran A, Suarez M, Rodríguez MA, Vinaixa M, Samino S, Arola L, Correig X, Yanes O. Assessment of compatibility between extraction methods for NMR- and LC/MS-based metabolomics. Anal Chem. 2012 Jul 17;84(14):5838-44. doi: 10.1021/ac3005567. Epub 2012 Jun 26. PMID: 22697410.)

Our choice was to accept one extraction method that could be readily implemented in future studies. We opted for a simple uniform approach to sample handling that could be implemented in the future.

5. In line 119, the reported injection volume of 2 ml appears unusually high for LC-UV analysis, and in line 123 the reconstitution volume of 30 ml may require clarification or correction.
**Reponse**: Thank you for pointing out the typos. They have been corrected to “2 µL” and “30 µL,” respectively. Upon further review of the method, we determined that the entire “Sample Normalization” step was not appropriate and has therefore been removed from the manuscript. The normalization was in fact carried out during the “Sample Preparation” step using the NovaMT Sample Normalization Kit.

6.The box plots in Figure 4 lack labeled y-axes and units
**Response**: Figure legends now indicate that the y-axis shows the relative intensities of ^12^C/^13^C ratios.

7. Line 268: How were these metabolites selected and were multiple testing corrections applied?

**Response**: The top 10 statistically performing metabolites for each of the three isotope groups were examined. These were correlated with Neutral Mass of the metabolite in Daltons, and the the Normalized Retention Time in seconds. These were cross-referenced with the Neutral Mass and Normalized Retention Time values in metabolite identification data for each isotope group to determine which ones were identified. Some top performing metabolites were not chemically identified. The results are below (I omitted the numbers which correlated with unidentified metabolites).

Amine-Phenol top 10

- 182.057_1503.9 [tier 2] 3,4-Dihydroxyphenylpropanoic acid
- 276.0946_212.6 [tier 1] Glutamyl-Glutamic acid
- 103.0625_655.9 [tier 2] N-ethylglycine

Carboxy top 10

- 153.0404_509.4 [tier 1] 3-Aminosalicylic acid
- 213.0086_439.4 [tier 3] Indoxyl sulfate

Hydroxyl top 10

- 104.0479_488.8 [tier 2] 4-Hydroxybutanoic acid

The "tier" refers to the level of confidence of chemical identification based on LC-MS data and references to multiple chemical isotope libraries, with tier 1 being the highest. tier 1 is "Positive Identification", tier 2 as "High Confidence Putative Identification", and tier 3 as "Putative Identification". 

8. It would be helpful to assess whether the observed performance differences between linear and non-linear SVM models are statistically significant. **Response**: New Table 4 shows the results of statistical comparison of linear vs nonliner SVM performance.

9. The authors should clearly discuss the limitations and weaknesses of the study, including the lack of external validation. **Response**: Study strengths and weaknesses have been added to the discussion. Strengths include enrollment of samples from multiple centers, limiting single site biases related to sample procurement, handling and storage. Censoring data at 3 years vs 5 years or longer likely includes a substantial majority (approximately 80%) but nor all potential metastases (Chin AI, Lam JS, Figlin RA, Belldegrun AS. Surveillance strategies for renal cell carcinoma patients following nephrectomy. Rev Urol. 2006 Winter;8(1):1-7. PMID: 16985554; PMCID: PMC1471767). We acknowledge that an external validation set of ccRCC was not analyzed and therefore limits the study.

10. Several metabolites reported as discriminative are not strictly endogenous (e.g., norcotinine, 3-aminosalicylic acid), or are strongly influenced by exogenous factors such as smoking, diet, microbiota, or renal function (e.g., indoxyl sulfate). The authors should clarify the origin of these compounds
**Response**: Brief comments about narcotinine and 3-aminosalicylic acid are added in the discussion. Additional comments are added in the discussion for indoxyl sulfate.

11. Several metabolites discussed as tumor-related markers are known to be strongly influenced by exogenous factors such as diet, smoking, microbiota, renal function, or medication use. This should be acknowledged more explicitly in the Discussion to avoid overinterpretation of their biological relevance.
**Response**: Additional comments in the Discussion are provided.

12. The manuscript uses the term "predict" metastasis, however the findings are more appropriately described as prognostic rather than predictive. This distinction should be clarified throughout the manuscript.
**Response:** Predict has been changed to prognostic in the title and throughout the text.

13. The manuscript does not clearly indicate which of the reported metabolites have been previously associated with ccRCC prognosis and which are proposed here as novel prognostic markers.

**Response**: New references are inserted in the introduction and discussion for reported metabolites previously associated with prognosis.

14. Authors should include representative extracted ion chromatograms for selected, ultimately identified metabolites in the supplementary materials.
**Response**: Thank you for the suggestion. We have added Supplemental Figure 1, which presents extracted ion chromatograms (EICs) of six representative identified metabolites frequently altered in ccRCC tumor samples, including lactate, malate, citrate, succinate, glutamyl-glutamic acid, and kynurenine, as examples. We added the following text in the manuscript: “Extracted ion chromatograms (EICs) of selected metabolites from a representative individual sample are provided in Supplemental Figure 1 as examples.”

15. Minor editorial issues:

- Line 129: "protol" should be corrected to "protocol." **Response:** Corrected.
- The capitalization of "tier" is inconsistent throughout the manuscript ("tier" vs. Tier") and should be standardized. **Response:** Corrected to tier.
- There are several inconsistencies in capitalization throughout the manuscript, including metabolite names and method names being capitalized mid-sentence. These should be standardized for consistency and readability. **Response**: Capitalization has been standardize
