# Supplementary material for: Metabolomic profiling of renal cell carcinoma to identify prognostic markers of metastasis
Source: PLoS One. 2026 Nov 11;21(11):e0361404. doi: 10.1371/journal.pone.0361404 (PMC13754214; doi:10.1371/journal.pone.0361404)
Supplement: Peer review history

# Peer review: Decision Letter 0

**DOI:** 10.1371/journal.pone.0361404.r001

**Authors:** Saki Raheem

1 Feb 2026

Dear Dr. Troyer,

Thank you for submitting your manuscript to PLOS ONE. After careful consideration, we feel that it has merit but does not fully meet PLOS ONE’s publication criteria as it currently stands. Therefore, we invite you to submit a revised version of the manuscript that addresses the points raised during the review process.

Please submit your revised manuscript by Mar 18 2026 11:59PM. If you will need more time than this to complete your revisions, please reply to this message or contact the journal office at plosone@plos.org. When you're ready to submit your revision, log on to https://www.editorialmanager.com/pone/ and select the 'Submissions Needing Revision' folder to locate your manuscript file.

- A letter that responds to each point raised by the academic editor and reviewer(s). You should upload this letter as a separate file labeled 'Response to Reviewers'.
- A marked-up copy of your manuscript that highlights changes made to the original version. You should upload this as a separate file labeled 'Revised Manuscript with Track Changes'.
- An unmarked version of your revised paper without tracked changes. You should upload this as a separate file labeled 'Manuscript'.

If you would like to make changes to your financial disclosure, please include your updated statement in your cover letter. Guidelines for resubmitting your figure files are available below the reviewer comments at the end of this letter.

If applicable, we recommend that you deposit your laboratory protocols in protocols.io to enhance the reproducibility of your results. Protocols.io assigns your protocol its own identifier (DOI) so that it can be cited independently in the future. For instructions see: https://journals.plos.org/plosone/s/submission-guidelines#loc-laboratory-protocols. Additionally, PLOS ONE offers an option for publishing peer-reviewed Lab Protocol articles, which describe protocols hosted on protocols.io. Read more information on sharing protocols at https://plos.org/protocols?utm_medium=editorial-email&utm_source=authorletters&utm_campaign=protocols.

We look forward to receiving your revised manuscript.

Kind regards,

Saki Raheem, PhD

Academic Editor

PLOS One

Journal Requirements:

When submitting your revision, we need you to address these additional requirements.

1. Please ensure that your manuscript meets PLOS ONE's style requirements, including those for file naming. The PLOS ONE style templates can be found at

https://journals.plos.org/plosone/s/file?id=wjVg/PLOSOne_formatting_sample_main_body.pdf and

https://journals.plos.org/plosone/s/file?id=ba62/PLOSOne_formatting_sample_title_authors_affiliations.pdf

2. Please note that PLOS One has specific guidelines on code sharing for submissions in which author-generated code underpins the findings in the manuscript. In these cases, we expect all author-generated code to be made available without restrictions upon publication of the work. Please review our guidelines at https://journals.plos.org/plosone/s/materials-and-software-sharing#loc-sharing-code and ensure that your code is shared in a way that follows best practice and facilitates reproducibility and reuse.

3. Thank you for stating the following in the Acknowledgments Section of your manuscript:

“1) NCI1R43CA228686-01A1 2) Provia Biologics 3) Eastern Virginia Medical School Biorepository 4) Urology of Virginia”

We note that you have provided funding information that is not currently declared in your Funding Statement. However, funding information should not appear in the Acknowledgments section or other areas of your manuscript. We will only publish funding information present in the Funding Statement section of the online submission form.

Please remove any funding-related text from the manuscript and let us know how you would like to update your Funding Statement. Currently, your Funding Statement reads as follows:

“Sources of Funding: 1) NCI1R43CA228686-01A1 2) Provia Biologics 3) Eastern Virginia Medical School Biorepository 4) Urology of Virginia; Coded specimens and de-identified clinical data elements were provided from three biorepositories for this study so that authors had no access to subject identifiers.  Day, month, and year when data were accessed for research purposes were as follows.  Alberta Cancer Research Biobank (https://www.acrb.ca/) 04/03/2020.  Old Dominion University Biorepository and Histology (Formerly Eastern Virginia Medical School Biorepository) 02/07/2020.  UT Health San Antonio Mays Cancer Center Biorepository (Formerly University of Texas Health Science Center San Antonio) 09/06/2019.  The research was conducted under IRB Protocol 17-08-NH-0168, Old Dominion University, formerly Eastern Virginia Medical School. “

Please include your amended statements within your cover letter; we will change the online submission form on your behalf.

4. We note that the grant information you provided in the ‘Funding Information’ and ‘Financial Disclosure’ sections do not match.

When you resubmit, please ensure that you provide the correct grant numbers for the awards you received for your study in the ‘Funding Information’ section.

5. Thank you for stating the following financial disclosure:

“Sources of Funding: 1) NCI1R43CA228686-01A1 2) Provia Biologics 3) Eastern Virginia Medical School Biorepository 4) Urology of Virginia; Coded specimens and de-identified clinical data elements were provided from three biorepositories for this study so that authors had no access to subject identifiers.  Day, month, and year when data were accessed for research purposes were as follows.  Alberta Cancer Research Biobank (https://www.acrb.ca/) 04/03/2020.  Old Dominion University Biorepository and Histology (Formerly Eastern Virginia Medical School Biorepository) 02/07/2020.  UT Health San Antonio Mays Cancer Center Biorepository (Formerly University of Texas Health Science Center San Antonio) 09/06/2019.  The research was conducted under IRB Protocol 17-08-NH-0168, Old Dominion University, formerly Eastern Virginia Medical School. “

Please state what role the funders took in the study.  If the funders had no role, please state: "The funders had no role in study design, data collection and analysis, decision to publish, or preparation of the manuscript."

If this statement is not correct you must amend it as needed.

Please include this amended Role of Funder statement in your cover letter; we will change the online submission form on your behalf.

6. When completing the data availability statement of the submission form, you indicated that you will make your data available on acceptance. We strongly recommend all authors decide on a data sharing plan before acceptance, as the process can be lengthy and hold up publication timelines. Please note that, though access restrictions are acceptable now, your entire data will need to be made freely accessible if your manuscript is accepted for publication. This policy applies to all data except where public deposition would breach compliance with the protocol approved by your research ethics board. If you are unable to adhere to our open data policy, please kindly revise your statement to explain your reasoning and we will seek the editor's input on an exemption. Please be assured that, once you have provided your new statement, the assessment of your exemption will not hold up the peer review process.

7. Please amend the manuscript submission data (via Edit Submission) to include author Ya-Chun Chen and Liam Spears.

8. Please amend your authorship list in your manuscript file to include author Ya-Chun Chan and Liam Spiers.

Reviewers' comments:

Reviewer's Responses to Questions

**Comments to the Author**

1. Is the manuscript technically sound, and do the data support the conclusions?

Reviewer #1: Yes

Reviewer #2: Partly

**********

2. Has the statistical analysis been performed appropriately and rigorously?

Reviewer #1: Yes

Reviewer #2: Yes

**********

3. Have the authors made all data underlying the findings in their manuscript fully available?

The PLOS Data policy

Reviewer #1: No

Reviewer #2: No

**********

4. Is the manuscript presented in an intelligible fashion and written in standard English?

Reviewer #1: Yes

Reviewer #2: Yes

**********

Reviewer #1: In this study the authors evaluated the metabolomic profiles of ccRCC that metastasized during a minimum follow-up period of 3 years versus those that did not. This is a very well-performed study with interesting results

I have some minor comments:

-ccRCC is essentially a metabolic disease characterized by a reprogramming of energetic metabolism (PMID: 40082108; PMID: 36960789; PMID: 30983433, PMID: 36430837,PMID: 36310399). In particular the metabolic flux through glycolysis is partitioned (PMID: 30538212; PMID: 29371925, PMID: 28933387, PMID: 25945836), and mitochondrial bioenergetics and OxPhox are impaired , as well as lipid metabolism (PMID: 29371925, PMID: 36430448). In addition, metabolic reprogramming plays an important role in chemoresistance and/or in determining the sensitivity of neoplastic cells to drugs (PMID: 41533164) .

These findings should be referenced and better discussed.

Reviewer #2: The manuscript investigates metabolomic profiles of primary ccRCC tumors to identify markers associated with metastatic progression using LC–MS–based chemical isotope labeling and machine learning approaches. The study addresses a clinically relevant question however several aspects require clarification and more cautious interpretation.

1. The Introduction currently lacks a clearly articulated statement of novelty and a concise review of the most relevant LC–MS–based metabolomic studies in RCC (e.g., DOI: 10.1038/s41598-019-52059-y, 10.1186/s12894-025-01767-x, 10.1016/j.eururo.2025.09.4148, 10.1007/s11306-025-02294-4, 10.3390/ijms232214360).

2. Key clinical information is missing, including tumor stage and grade, tumor size, presence of tumor necrosis, type of surgical procedure, postoperative treatment, concomitant medications, renal function and relevant comorbidities.

3. The terminology used to describe the study groups is confusing. Please clarify that “controls” refer to non-metastatic ccRCC patients rather than healthy individuals, and clearly distinguish patient groups (metastatic vs. non-metastatic tumors) from the adjacent normal tissue samples included in the analysis.

4. Why was extraction performed for 24 h at room temperature rather than using conventional cold extraction protocols?

5. In line 119, the reported injection volume of 2 mL appears unusually high for LC-UV analysis, and in line 123 the reconstitution volume of 30 mL may require clarification or correction.

6. The box plots in Figure 4 lack labeled y-axes and units

7. Line 268: How were these metabolites selected and were multiple testing corrections applied?

8. It would be helpful to assess whether the observed performance differences between linear and non-linear SVM models are statistically significant.

9. The authors should clearly discuss the limitations and weaknesses of the study, including the lack of external validation.

10. Several metabolites reported as discriminative are not strictly endogenous (e.g., norcotinine, 3-aminosalicylic acid), or are strongly influenced by exogenous factors such as smoking, diet, microbiota, or renal function (e.g., indoxyl sulfate). The authors should clarify the origin of these compounds

11. Several metabolites discussed as tumor-related markers are known to be strongly influenced by exogenous factors such as diet, smoking, microbiota, renal function, or medication use. This should be acknowledged more explicitly in the

Discussion to avoid overinterpretation of their biological relevance.

12. The manuscript uses the term “predict” metastasis, however the findings are more appropriately described as prognostic rather than predictive. This distinction should be clarified throughout the manuscript.

13. The manuscript does not clearly indicate which of the reported metabolites have been previously associated with ccRCC prognosis and which are proposed here as novel prognostic markers.

14. Authors should include representative extracted ion chromatograms for selected, ultimately identified metabolites in the supplementary materials.

15. Minor editorial issues:

- Line 129: “protol” should be corrected to “protocol.”

- The capitalization of “tier” is inconsistent throughout the manuscript (“tier” vs. Tier”) and should be standardized.

- There are several inconsistencies in capitalization throughout the manuscript, including metabolite names and method names being capitalized mid-sentence. These should be standardized for consistency and readability.

**********

what does this mean?). If published, this will include your full peer review and any attached files.

If you choose “no”, your identity will remain anonymous but your review may still be made public.

**Do you want your identity to be public for this peer review?**  For information about this choice, including consent withdrawal, please see our Privacy Policy

Reviewer #1: No

Reviewer #2: No

**********

[NOTE: If reviewer comments were submitted as an attachment file, they will be attached to this email and accessible via the submission site. Please log into your account, locate the manuscript record, and check for the action link "View Attachments". If this link does not appear, there are no attachment files.]

To ensure your figures meet our technical requirements, please review our figure guidelines: https://journals.plos.org/plosone/s/figures

You may also use PLOS’s free figure tool, NAAS, to help you prepare publication quality figures: https://journals.plos.org/plosone/s/figures#loc-tools-for-figure-preparation.

NAAS will assess whether your figures meet our technical requirements by comparing each figure against our figure specifications.

# Author comment: Author response to Decision Letter 1

**DOI:** 10.1371/journal.pone.0361404.r002

11 Sep 2026

We are grateful for the reviewers comments. Retrievable clinical data elements are included in the supplementary tables 1 and 2.

Attachment Submitted filename: Plos1 Response to reviewers 07.31.26.docx

# Peer review: Decision Letter 1

**DOI:** 10.1371/journal.pone.0361404.r003

**Authors:** Saki Raheem

12 Oct 2026

Metabolomic Profiling of Renal Cell Carcinoma to Identify Prognostic Markers of  Metastasis

PONE-D-25-69184R1

Dear Dr. Troyer,

We’re pleased to inform you that your manuscript has been judged scientifically suitable for publication and will be formally accepted for publication once it meets all outstanding technical requirements.

Within one week, you’ll receive an e-mail detailing the required amendments. When these have been addressed, you’ll receive a formal acceptance letter and your manuscript will be scheduled for publication.

An invoice will be generated when your article is formally accepted. Please note, if your institution has a publishing partnership with PLOS and your article meets the relevant criteria, all or part of your publication costs will be covered. Please make sure your user information is up-to-date by logging into Editorial Manager at Editorial Manager® and clicking the ‘Update My Information' link at the top of the page. For questions related to billing, please contact billing support.

If your institution or institutions have a press office, please notify them about your upcoming paper to help maximize its impact. If they’ll be preparing press materials, please inform our press team as soon as possible -- no later than 48 hours after receiving the formal acceptance. Your manuscript will remain under strict press embargo until 2 pm Eastern Time on the date of publication. For more information, please contact onepress@plos.org.

Kind regards,

Saki Raheem, PhD

Academic Editor

PLOS One

Reviewers' comments:

Reviewer's Responses to Questions

**Comments to the Author**

Reviewer #1: All comments have been addressed

**********

2. Is the manuscript technically sound, and do the data support the conclusions?

Reviewer #1: Yes

**********

3. Has the statistical analysis been performed appropriately and rigorously?

Reviewer #1: Yes

**********

4. Have the authors made all data underlying the findings in their manuscript fully available?

The PLOS Data policy

Reviewer #1: Yes

**********

5. Is the manuscript presented in an intelligible fashion and written in standard English?

Reviewer #1: Yes

**********

Reviewer #1: (No Response)

**********

what does this mean?). If published, this will include your full peer review and any attached files.

If you choose “no”, your identity will remain anonymous but your review may still be made public.

**Do you want your identity to be public for this peer review?**  For information about this choice, including consent withdrawal, please see our Privacy Policy

Reviewer #1: No

**********

# Editor report: Acceptance letter

**DOI:** 10.1371/journal.pone.0361404.r004

**Authors:** Saki Raheem

PONE-D-25-69184R1

PLOS One

Dear Dr. Troyer,

I'm pleased to inform you that your manuscript has been deemed suitable for publication in PLOS One. Congratulations! Your manuscript is now being handed over to our production team.

At this stage, our production department will prepare your paper for publication. This includes ensuring the following:

* All references, tables, and figures are properly cited

* All relevant supporting information is included in the manuscript submission,

* There are no issues that prevent the paper from being properly typeset

You will receive further instructions from the production team, including instructions on how to review your proof when it is ready. Please keep in mind that we are working through a large volume of accepted articles, so please give us a few days to review your paper and let you know the next and final steps.

Lastly, if your institution or institutions have a press office, please let them know about your upcoming paper now to help maximize its impact. If they'll be preparing press materials, please inform our press team within the next 48 hours. Your manuscript will remain under strict press embargo until 2 pm Eastern Time on the date of publication. For more information, please contact onepress@plos.org.

You will receive an invoice from PLOS for your publication fee after your manuscript has reached the completed accept phase. If you receive an email requesting payment before acceptance or for any other service, this may be a phishing scheme. Learn how to identify phishing emails and protect your accounts at https://explore.plos.org/phishing.

If we can help with anything else, please email us at customercare@plos.org.

Thank you for submitting your work to PLOS One and supporting open access.

Kind regards,

PLOS One Editorial Office Staff

on behalf of

Dr. Saki Raheem

Academic Editor

PLOS One
